# Therapeutic Potential of Certain Terpenoids as Anticancer Agents: A Scoping Review

**DOI:** 10.3390/cancers14051100

**Published:** 2022-02-22

**Authors:** Sareh Kamran, Ajantha Sinniah, Mahfoudh A. M. Abdulghani, Mohammed Abdullah Alshawsh

**Affiliations:** 1 Department of Pharmacology, Faculty of Medicine, University of Malaya, Kuala Lumpur 50603, Malaysia; s2101030@siswa.um.edu.my (S.K.); ajantha.sinniah@um.edu.my (A.S.); 2 Department of Pharmacology and Toxicology, Unaizah College of Pharmacy, Qassim University, Al Qassim 51911, Saudi Arabia; ma.abdulghani@qu.edu.sa

**Keywords:** cancer, terpenoids, molecular mechanism, terpenes, chemosensitization

## Abstract

**Simple Summary:**

Terpenoids are bioactive compounds with a variety of pharmacological activities, including anticancer activity. This review paper aimed to focus on the anticancer activities of certain terpenoids, including their molecular mechanism, signaling pathways and clinical trials. In addition, this review provides insight and future directions to the development of some terpenoids as potential anticancer agents.

**Abstract:**

Cancer is a life-threatening disease and is considered to be among the leading causes of death worldwide. Chemoresistance, severe toxicity, relapse and metastasis are the major obstacles in cancer therapy. Therefore, introducing new therapeutic agents for cancer remains a priority to increase the range of effective treatments. Terpenoids, a large group of secondary metabolites, are derived from plant sources and are composed of several isoprene units. The high diversity of terpenoids has drawn attention to their potential anticancer and pharmacological activities. Some terpenoids exhibit an anticancer effect by triggering various stages of cancer progression, for example, suppressing the early stage of tumorigenesis via induction of cell cycle arrest, inhibiting cancer cell differentiation and activating apoptosis. At the late stage of cancer development, certain terpenoids are able to inhibit angiogenesis and metastasis via modulation of different intracellular signaling pathways. Significant progress in the identification of the mechanism of action and signaling pathways through which terpenoids exert their anticancer effects has been highlighted. Hence, in this review, the anticancer activities of twenty-five terpenoids are discussed in detail. In addition, this review provides insights on the current clinical trials and future directions towards the development of certain terpenoids as potential anticancer agents.

## 1. Introduction

Globally, cancer is a major death root. The cancer burden is described in terms of incidence and mortality. An update on the worldwide cancer burden according to GLOBOCAN 2020 provides an estimation of cancer incidence and mortality produced by the International Agency for Research on Cancer (IARC). Globally, 19.3 million recent cancer cases and nearly 10 million cancer-related mortality were reported in 2020. Female breast cancer as the most common cancer was estimated with 2.3 million (11.7%) new cases, followed by lung cancer (11.4%), colorectal cancer (10.0%), prostate cancer (7.3%) and stomach cancer (5.6%). In terms of mortality, lung cancer was estimated to be the highest leading cause of death with 1.8 million deaths (18%), followed by colorectal cancer (9.4%), liver (8.3%), stomach (7.7%) and female breast cancer (6.9%). Global cancer incidence is predicted to be 28.4 million cases in the year 2040, which indicates a 47% growth from 2020 [1].

Global cancer statistics in 2018 anticipated 18.1 million new cancer cases and 9.6 million deaths, with lung cancer being the major cause of mortality followed by stomach and liver cancer in males, while in females, breast cancer is the most frequent cancer and the leading cause of mortality followed by lung and colorectal cancer. Esophagus, pancreas and prostate cancers showed a lower mortality rate in comparison to the other cancer types [2].

Cancer is a condition that results from genetic mutations, which promote the uncontrollable division of cells either locally or metastatically [3]. This mutation appears as a result of exogenous or endogenous DNA damage. In normal conditions, cells that respond to this damage would stimulate a repair system that involves cell cycle arrest to suppress subsequent damage. P53 is a main responsive tumor suppressor gene that functions to control cell cycle arrest and induces apoptosis if necessary [4]. This process prevents the transmission of mutations to the next generations of cells [5]. However, when this mechanism is disrupted, cancer cell mutation occurs rapidly, leading to tumor development [6]. Under such conditions, several studies suggested that some terpenoids have apoptotic potential against cancer cells [7,8,9].

In this review, the literature on the anticancer activities of twenty-five terpenoids (Table 1) is critically examined. The molecular mechanisms and signaling pathways through which each terpenoid exerts its therapeutic effects are discussed in depth. Effective concentrations of certain terpenoids and their targets based on IC_50_ values or tested range of concentrations against different cancer cell lines are shown as supplementary data (Table S1).

## 2. Classification of Terpenoids

Terpenoids are the largest and most widely diverse group of naturally occurring phytoconstituents. They produce the fragrance, taste and pigmentation of plants. Their classification is based on the number of carbons formed by isoprene units they contain. These isoprene units are the building blocks of the terpenoids, which is a gaseous hydrocarbon with a molecular formula of C_5_H_8._ Table 2 shows the classification of the naturally occurring terpenoids [206].

## 3. Mechanism of Action of Selected Terpenoids against Cancer Progression

### 3.1. Monoterpenoids

#### 3.1.1. Thymol

Thymol induces an anticancer effect in acute promyelocytic leukemia cells (HL-60) by arresting the cell cycle at G0/G1 phase and mitochondrial depolarization. The cytotoxic effect of thymol is through reactive oxygen species (ROS) production followed by mitochondrial membrane deterioration. Thymol also can increase the expression of Bax protein and reduce Bcl-2 protein, leading to the activation of caspases and apoptosis [10]. Moreover, thymol induced lipid degeneration, mitochondrial depolarization, nucleolar segregation and apoptosis in Caco-2 colorectal adenocarcinoma cells. Although the ultrastructural study showed cytotoxicity of thymol against cancer cells, further studies are required to identify the mechanisms that may be involved in this cytotoxicity [12]. A similar effect of thymol was also observed in AGC human gastric carcinoma cells, where the intrinsic apoptosis pathway and ROS generation resulted in cell death [13].

Similarly, thymol induced apoptosis in bladder cancer cells (T24, SW780, J82) through caspases activation [15]. Conversely, thymol in a 7-day treatment study demonstrated an anti-apoptotic response via the Bcl-2/Bax pathway [11]. Thymol-induced mitochondrial damage was also observed in oral squamous carcinoma cells (OSCC) and cervical cancer cells in a xenograft nude mice model [14]. In addition, thymol exerted its anticancer effect in human T lymphocyte Jurkat cells (JM) by playing an immunomodulatory role on T-cell activity via reducing interleukin-2 (IL-2) and interferon-gamma (IFN-_Y_) production. The proposed mechanism of action of thymol is due to the down-regulation of activator protein 1 (AP-1) and nuclear factor of activated T-cell (NFAT-2) as a transcription factor [16].

**Figure 1:**
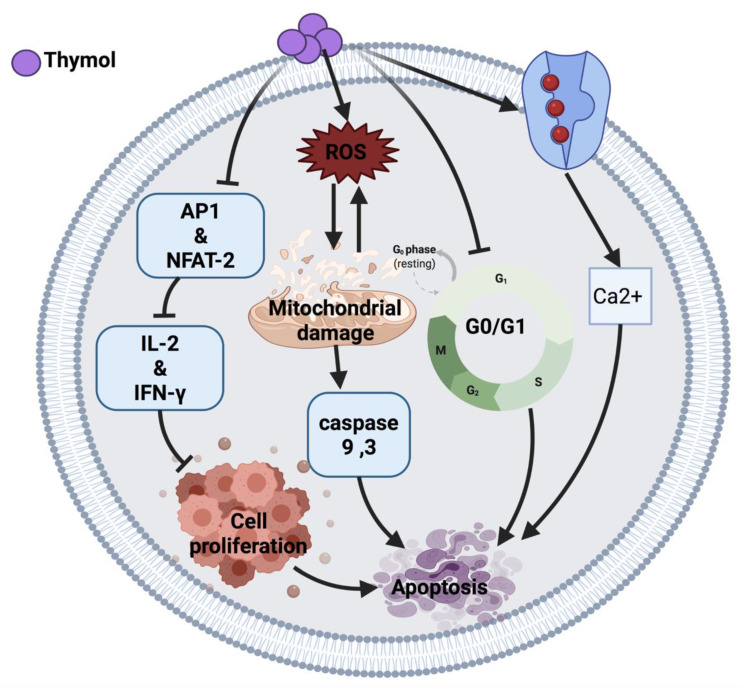
Molecular mechanisms involved in the effect of thymol on cancer cells. Ca^2+^: calcium ion, ROS: reactive oxygen species, AP1: activator protein 1, NFAT-2: nuclear factor of activated T cell, IL-2: interleukin-2, IFN-_Y_: interferon-gamma.

Thymol exerts its anticancer effect by inducing phospholipase C-dependent Ca^2+^ secretion from the endoplasmic reticulum and Ca^2+^ entry through store-operated Ca^2+^ channels, which may activate apoptosis in MG63 human osteosarcoma cells [17], DBTRG-05MG human glioblastoma cells [18] and PC3 human prostate cancer cells [19]. Moreover, thymol-induced Ca^2+^ secretion induces ROS generation, thus the exact mechanism of cell death is unclear [17]. Figure 1 illustrates an overview of the possible anticancer mechanisms of thymol.

#### 3.1.2. Menthol

According to Lin et al., the inhibitory molecular mechanism of menthol in SNU-5 human gastric cancer cells triggered the reduction of topoisomerase I and II followed by DNA damage and cell death. Eventually, targeting the suppression of topoisomerases could be a potential therapeutic strategy to be used in combination with other clinical approaches [20]. Another study has shown that 1alpha, 25-dihydroxyvitamin D3 (1α,25(OH)_2_D_3_) in combination with menthol might enhance the anticancer effect against prostate cancer cells via targeting the reduction of Bcl-2 protein expression and suppression of p21. Moreover, in other types of cancer, the 24-hydroxylase enzyme, which is a 1α,25(OH)_2_D_3_ catabolic enzyme, was shown to be up-regulated; this led to the inactivation of 1α,25(OH)_2_D_3_ and suggests a tumor escaping mechanism. Moreover, the effect of menthol on metabolic enzymes could be a challengeable approach in drug discovery [21]. It has been reported that cancer induces the efflux of Ca^2+^ from bones into the bloodstream, hence activating a calcium-permeable channel (TRPM8) resulting in cell death [23]. Menthol, in bladder cancer cell line (T24) induced mitochondrial membrane depolarization via increasing the Ca^2+^ level through the overexpression of TRPM8 in T24 cells leading to cancer cell death [22].

**Figure 2:**
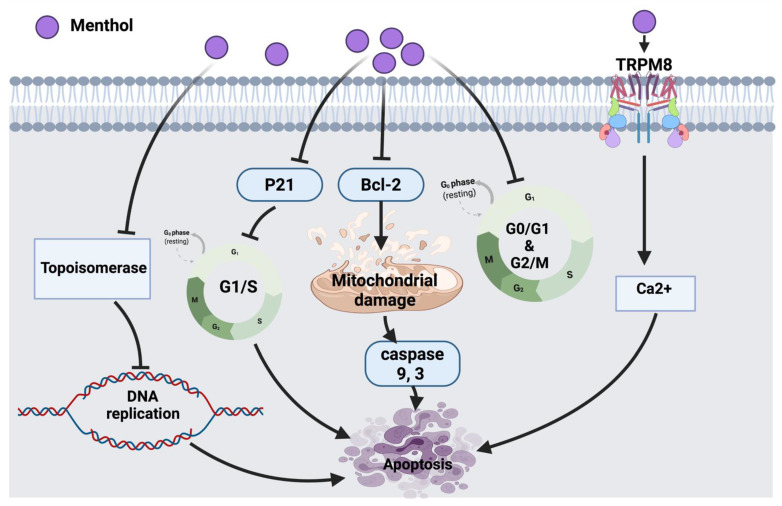
Molecular mechanisms involved in the effect of menthol on cancer cells. Bcl-2: B-cell lymphoma 2, TRPM8: transient receptor potential cation channel subfamily member 8, Ca^2+^: calcium ion, p21: cyclin-dependent kinase inhibitor.

Menthol also arrests cell cycles in androgen-independent DU145 prostate cancer cells at the G_0_/G_1_ phase by down-regulating CDK2, 4, 6 [24]. Another study that tested PC3 prostate cancer cells showed that menthol has the potential to arrest the cell cycle at the G2/M phase by down-regulating the downstream signaling of polo-like kinase 1 (PLK1). The outcomes provide a pharmacological basis for future investigations of menthol against other cancer cells [25]. The overall anticancer molecular mechanism of menthol is illustrated in Figure 2.

#### 3.1.3. Auraptene

Auraptene arrests the cell cycle and induces apoptosis in SNU-1 human gastric cancer cells via mitochondrial membrane damage. Auraptene was shown to up-regulate p53 tumor suppressor protein and decrease cyclin D1 levels; therefore, arresting cell progression through the G1 phase, leading to cell death. Auraptene also inhibited cell proliferation by down-regulating the mTOR signaling pathway and its downstream activity. Although this suggested anticancer mechanism of auraptene triggered mTOR inhibition, indirect activation of mTOR-rictor is a currently unknown mechanism that could be further investigated [27]. The synergistic role of auraptene together with anticancer drugs (cisplatin, paclitaxel, 5-fluorouracil) was studied on KYSE30 esophageal carcinoma cells. Auraptene enhanced the anticancer drug efflux transporters via interaction with the P-gp efflux pump, which increases the drug’s toxic effects inside the cancer cells. Auraptene also attenuates the malignant properties of cancer cells through activating p53 and p21 overexpression and down-regulation of cancer stem cells (CSC) markers, such as CD44 and BMI-1 [26].

Moreover, the effect of auraptene on human cervical cancer cells (HeLa cell) and ovarian cancer cells (A2780) showed growth-inhibitory effects, suppression of cell migration and metastasis. The possible anticancer effect of auraptene in these cell lines was suggested due to a decline in the expression levels of matrix metalloproteinases (MMP-2 and MMP-9) [28]. Auraptene also inhibits β-catenin T-cell factor (TCF) function in colorectal cancer cells and suppresses the overexpression of c-Myc proto-oncogene. In addition, auraptene treatment showed cell growth inhibition and G2/M phase arrest in Caco-2 human colorectal carcinoma and DLD-1 colorectal adenocarcinoma cells. This monoterpenoid has been shown to inhibit β-catenin/TCF activity in Caco-2 cells and enhance its activity in DLD-1 cells. This contradiction in the mechanism of auraptene action indicates that the cell growth inhibition of colorectal cancer cells by auraptene may be independent of the targeting of β-catenin-TCF signaling [30].

Another study suggested the inhibitory effect of auraptene in azoxymethane (AOM) induced colorectal preneoplastic lesions in mice. Auraptene significantly inhibited the formation of aberrant crypt foci (cancer markers), β-catenin-accumulated crypt and cell proliferation, as well as increased apoptosis [29]. Similarly, auraptene displayed an inhibitory effect on AOM-induced colon carcinogenesis in mice by reducing proliferating cell nuclear antigen (PCNA) and increasing DNA fragmentation. Moreover, auraptene showed the potential to ameliorate pro-inflammatory cytokines suggesting that this compound exerts its inhibitory effect against cancer through modulating inflammation. It is worth mentioning that enhancing auraptene bioavailability and improving tumor targeting is necessary to increase the chemo-preventive efficacy of auraptene [31].

**Figure 3:**
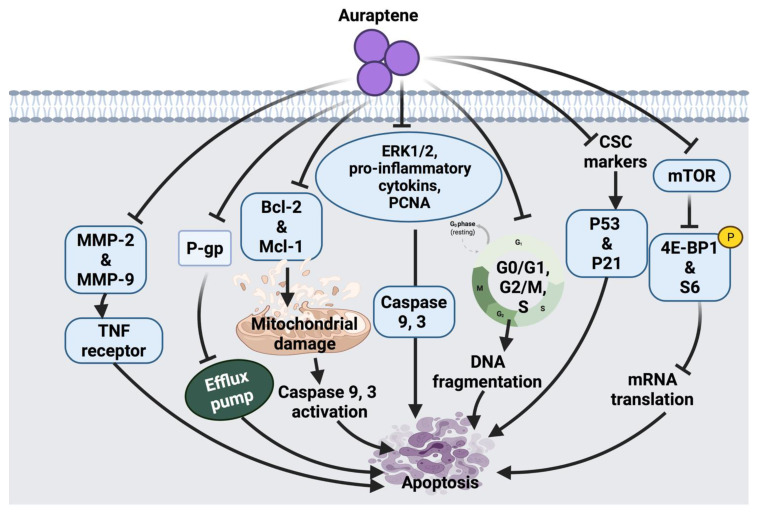
Molecular mechanisms involved in the effect of auraptene on cancer cells. MMP-2: matrix metalloproteinase-2, MMP-9: matrix metalloproteinase-9, TNF: tumor necrosis factor, P-gp: P-glycoprotein, Bcl-2: B-cell lymphoma 2, Mcl-1: apoptosis regulator, ERK1/2: extracellular signal-regulated kinases, PCNA: proliferating cell nuclear antigen, CSC: cancer stem cell surface, p53: tumor protein, p21: cyclin-dependent kinase inhibitor, mTOR: mammalian target of rapamycin, 4E-BP1: eukaryotic translation initiation factor 4E-binding protein 1.

Auraptene arrests MCF-7 mammary adenocarcinoma cells in the S phase through alteration of some of the transcription genes involved in cell cycle progression, including the down-regulation of cyclin D1 protein expression level and the suppression of insulin-like growth factor-1 (IGF-1). Similarly, the chemo-preventive effect of auraptene in human breast cancer cells (MCF-7 and MDA-MB-231) was reported due to the inhibition of cyclin D1 expression and cell cycle progression. Since auraptene inhibitory effect on cell cycle triggered via several pathways, further investigation on the potential synergistic effect of this compound in combination with other chemotherapeutic agents was suggested to be carried out [32]. Moreover, the ability of auraptene to suppress the tumor incidence and multiplicity was found to be significant at week 16 after treatment; however, toward the end of the experiment, both effects were not significant. This indicates that auraptene may delay the progression of tumor growth only during the early stages of cancer; therefore, future investigation at different time points of tumor development will provide more information regarding the anti-tumor effect of auraptene [33]. In PC3 and DU145 cancer cells, the possible anticancer mechanism of auraptene was suggested to be through Mcl-1-mediated caspase activation. Auraptene activated caspase 9 and 3, enhanced expression of Bax and reduced expression of Bcl-2 and Mcl-1 [34]. A summarized overview of the molecular mechanism of auraptene against cancer cells is shown in Figure 3.

#### 3.1.4. D-Limonene

**Figure 4:**
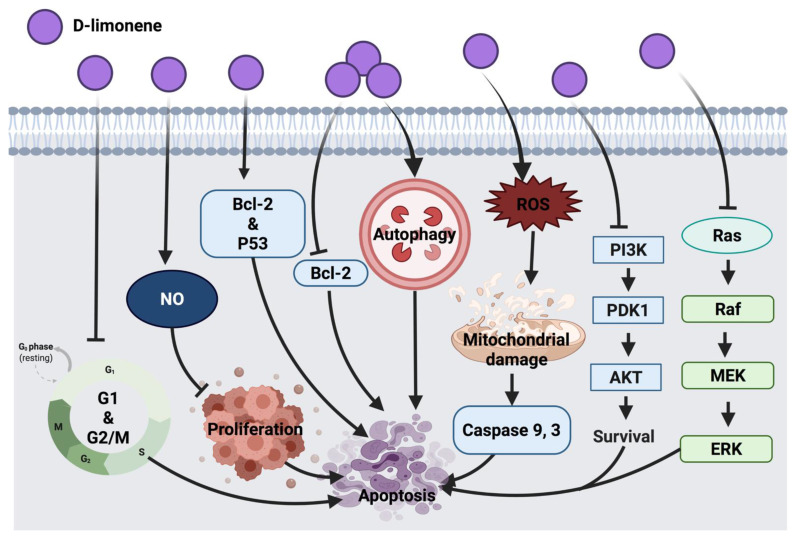
Molecular mechanisms involved in the effect of D-limonene on cancer cells. Bcl-2: B-cell lymphoma 2, Bax: Bcl-2 associated X protein, p53: tumor protein, NO: nitric oxide, ROS: reactive oxygen species, PI3K: phosphoinositide 3-kinase, PDK1: 3-phosphoinositide-dependent kinase 1, Akt: protein kinase B, Ras: rat sarcoma, Raf: rapidly accelerated fibrosarcoma, MEK: mitogen-activated protein kinase, ERK: extracellular signal-regulated kinases.

The immunomodulatory effect of D-limonene through macrophages was suggested to inhibit cell proliferation via enhancing nitric oxide (NO) production, which indicates that D-limonene anticancer activity is associated with macrophages activation in BALB/c mice with lymphoma cells. Since D-limonene was suggested to modulate immune response via macrophages, future consideration would be the investigation of mechanisms involved in macrophages activation and innate and specific immunity [37]. D-limonene inhibited HL-60 leukemia cell proliferation through the down-regulation of Bcl-2 and alteration of the p53 gene while enhancing the expression of the pro-apoptotic Bax gene [38]. In lung cancer (A549 and H1299), this compound exerted autophagy response via the cleavage of Atg5 pro-apoptotic agent and through the interaction with Bcl-XL transmembrane protein in mitochondria. This resulted in the activation of caspases involved in apoptosis and cell death. Partial involvement of Atg5 was speculated in D-limonene-induced apoptosis, thus a deep investigation into the mechanism underlying the role of autophagy in D-limonene-induced apoptosis could be considered in the future [35]. Furthermore, the synergistic effect of D-limonene with berberine was evaluated in MGC803 human gastric cancer cells, where it stimulated ROS generation intracellularly, facilitated mitochondrial damage, up-regulated caspase 3 activity and down-regulated Bcl-2 protein expression and mediated cell death. Additionally, D-limonene’s synergistic effect with berberine showed cell cycle suppression at G_1_ and G_2_/M phases and apoptosis promotion through the intrinsic mitochondrial pathway. However, additional investigations are required to elucidate mechanistic interactions of the combined drugs [39]. Skin tumor treatment with D-limonene inhibited the expression of Ras, Raf, MEK and ERK1/2. This anticancer mechanism was detected along with the down-regulation of Bcl-2 and up-regulation of Bax proteins [40]. PI3K/AKT pathway inhibition was detected as an anticancer mechanism of D-limonene in LS174T colon cancer cells [36]. Further investigation in other colon cancer cell lines could be considered in future studies. Figure 4 represents an overview of the anticancer molecular mechanism of D-limonene.

#### 3.1.5. Perillic Acid

Perillic acid in non-small cell-lung cancer (A549) suggested a dose-dependent cytotoxic response, caused cell cycle arrest and apoptosis, as well as marked elevation of Bax, p21 and caspase 3 expression level. However, it was speculated that this monoterpenoid targets multiple signaling sites and not only the apoptosis signaling pathway, including the inhibition of DNA repair. Therefore, further investigations of other possible mechanistic pathways are required [41]. The anticancer effect of perillic acid triggered over-expression of p21 and down-regulation of CDK1 and CDK4 in HCT116 colon cancer cells [42]. Although this effect was suggested in colon cancer cells, only the wild-type (p53+) cell line was tested in this experiment. Further investigation can be conducted on null p53 colon cancer cells.

#### 3.1.6. Ascaridole

In the investigation of the anticancer mechanism of ascaridole, oxidative DNA damage and G2/M phase cell cycle arrest were proposed in NER (nucleotide excision repair, main pathway to remove DNA damage)-deficient cell lines, such as XP3BE (XPC-deficient) and GM10902 (ERCC6 deficient). The cytotoxicity of ascaridole against NER-deficient cells was compared to repair-proficient cells, hence ascaridole showed selective cytotoxicity toward NER-deficient cells at concentrations much lower than that toward repair-proficient cells. This suggested the involvement of other plausible mechanisms, such as synthetic lethal interactions [43]. Since this effect was observed only at cytotoxic concentration, the causative role of ascaridole-induced oxidative stress in cellular death warrants further investigation. In the HL-60 human leukemia cell line, HCT-8 human colon cancer, SF-295 brain tumor cell line and MDA-MB-435 human breast cancer cells, ascaridole as a natural endoperoxide mediated the generation of ROS and interacted with intracellular targets, which enhanced apoptosis and cell death [44].

#### 3.1.7. Carvacrol

Carvacrol exerts its tumor inhibitory effect in HCT116 human colon cancer cells by blocking MMP-2 and MMP-9, a similar mechanism exerted by auraptene on cancer cells. Treatment of HCT-116 and LoVo human colon cancer cells with this compound also blocked cyclin B1 expression and G_2_/M phase progression. This compound was shown to reduce the expression level of anti-apoptotic proteins (Bcl-2 and phosphorylation of Akt) and increase apoptotic stimulating agents (Bax and JNK phosphorylation). It also showed an inhibitory role in the survival of signaling pathways, such as MAPK and PI3K/Akt; thus, suppressing cell proliferation. One of the limitations of this study is the high dosage of carvacrol used to trigger apoptosis. Indeed, 100–700 μM/L is relatively high compared to other anticancer agents [45].

In AGS human gastric adenocarcinoma cells, carvacrol was shown to initiate apoptosis and ROS generation leading to cell death; however, only at higher concentrations (tested concentration range: 0–100 μM/L) could carvacrol exert such an effect [46]. In MCF-7 breast cancer cells, this compound was shown to induce cell death by activating the apoptosis pathway via modulating p53, Bcl-2 and Bax proteins expression and stimulated caspases 9, 3 activations and DNA fragmentation [55]. Another study described the similar apoptotic molecular mechanism of carvacrol in HepG-2 (human hepatocarcinoma) cells [56]. Similar to auraptene, carvacrol in an in vivo study showed the potential to inhibit tumor development by suppressing aberrant crypt foci formation in colorectal cancer cells (CRC) through enhancing colonic lipid peroxidation and antioxidant enzymes activity. In this study, the maximum inhibitory effect against DMH-induced colon carcinogenesis was mediated by an intermediate dose of 40 mg/kg in comparison to lower (30 mg/kg) and higher (80 mg/kg) doses. At a lower dose, carvacrol concentration was not enough to alleviate DMH-induced damage, while at high concentration, carvacrol may change its antioxidant activity followed by a decrease in its potential [57]. Therefore, a long-term study on biochemical and molecular aspects is suggested to pinpoint the main mechanism by which carvacrol induces its anticancer effect against colon carcinogenesis. The cytotoxic effect of carvacrol in A549 lung carcinoma epithelial cells was proven by targeting cytoplasmic blebs formation and DNA damage. However, this effect was shown in cells treated with 500–1000 μM/l carvacrol, which remains a high dose range compared to other anticancer drugs [58]. In addition, carvacrol in LNCaP [59], MDA-MB-231 and U87 [207,208] and HEp-2 (human epithelial type 2) [62] cancer cells mediated cell death via mitochondrial membrane depolarization and apoptosis induction.

Moreover, carvacrol induced the down-regulation of IL-6, which resulted in the reduced expression of pSTAT3, pERK1/2 and pAkt cellular signaling proteins and resulted in the inhibition of cell proliferation, viability and invasion of PC3 cells. In this study, the authors did not investigate other possible mechanisms involved in the effect of carvacrol against PC3, such as apoptosis, necrosis, NF-_K_B, cleavage of caspase 3, Bax and Bcl-2 protein expression. This could be suggested in future research [47].

In DU145 human prostate cancer cells, carvacrol induced ROS-linked apoptosis with cell cycle suppression at G_0_/G1 phase. Further study is underway to explore the molecular mechanism involved in the anticancer effect of carvacrol [48]. A recent study showed that the inhibitory effect of carvacrol on the cell cycle is associated with the down-regulation of cyclin D1 and CDK4 and the up-regulation of suppressor protein p21 in androgen-independent PC-3 human prostate cancer. Aside from this, carvacrol blocked Notch-1 and Jagged-1 protein expression, resulting in the inhibition of the Notch signaling pathway [49]. In another study, CO25 myoblast cells treated with carvacrol showed cell cycle arrest at the G_0_/G1 phase; this suggested the apoptotic effect of carvacrol and suppression of DNA synthesis. Prenylation of Ras enables its association with plasma membrane followed by its oncogenic activity, the impairment in the prenylation of Ras was suggested to count for the anti-tumor activity of carvacrol. In addition to Ras, prenylated proteins TC21, Rho and PRL-PTP-CAAX tyrosine phosphatases are suggested to be oncogenic; therefore, these proteins could be taken as cellular targets for carvacrol in the future [50]. In a diethylnitrosamine (DEN)-induced hepatocellular carcinoma animal model, carvacrol exerted its effect by scavenging free radicals and inhibiting lipid peroxidation, leading to prevent hepatic tumor formation. However, further investigation on the effects of carvacrol as an anticancer candidate still has a long way to go [51].

**Figure 5:**
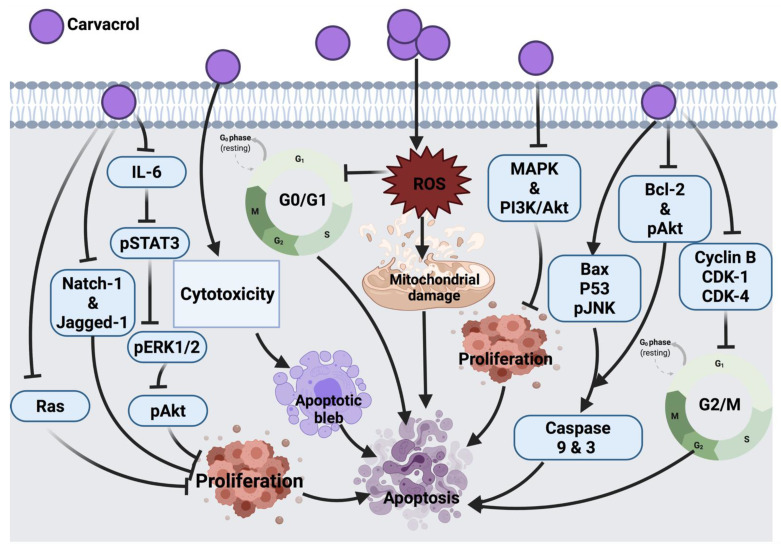
Molecular mechanisms involved in the effect of carvacrol on cancer cells. IL-6: interleukin 6, pSTAT3: phosphorylated signal transducer and activator of transcription 3, pERK1/2: phosphorylated extracellular signal-regulated kinase 2, Natch-1 and Jagged-1: oncogenes, Ras: rat sarcoma, pAkt: phosphorylated Protein kinase B, ROS: reactive oxygen species, MAPK: mitogen-activated protein kinase, PI3K/Akt: Phosphoinositide 3-kinase/ Protein kinase B, Bax: Bcl-2 associated X protein, p53: tumor protein, pJNK: phosphorylated c-Jun N-terminal kinase, Bcl-2: B-cell lymphoma 2, CDK-1: cyclin-dependent kinase 1, CDK-4: cyclin-dependent kinase 4.

Furthermore, a dose-dependent cytotoxic effect was also reported in the P815 mastocytoma cell line upon carvacrol treatment [52]. Cell cycle arrest at the S phase was induced as a result of carvacrol induction in P815, CEM, K-562, MCF-7 and MCF-7 breast tumor cells. However, the link between the molecular structure and cytotoxic effect of carvacrol remains to be investigated. Moreover, the type of cells might be involved in their differential sensitivity to carvacrol. Whether P53 mutation in CEM and P-815 selected cell lines is associated with their sensitivity to carvacrol warrants further investigations [53]. Another study that tested the carvacrol effect against DMBA-induced lung tumor in an animal model showed a significant anti-tumor effect of carvacrol; however, the mechanism of anti-tumor activity of this compound was not assessed. As a future consideration, a link between calcium metabolism and the anti-tumor effect of carvacrol was suggested [54]. The anticancer molecular mechanism of carvacrol against different cancer cells is illustrated in Figure 5.

#### 3.1.8. Thymoquinone

Findings from Rooney and collaborators [209] revealed that HEp-2 cells are the most sensitive to the cytotoxic effect of thymoquinone compared to A-549, HT-29 and MIA PaCa-2 (pancreas carcinoma) cell lines. In their study, the anticancer mechanism of thymoquinone was demonstrated through targeted mitochondrial glutathione (GSH) depletion, activation of caspase 3 and apoptosis. Although thymoquinone-treated HEp-2 cells (25 and 50 μM/L) induced apoptosis, at higher concentrations of thymoquinone (75 μM/L), necrosis was the dominant cellular death mechanism [209]. In another study, black cumin essential oil showed anticancer activity against A549 and DLD-1 (colon adenocarcinoma) cancer cells; this activity was claimed due to the effect of thymoquinone. However, no further investigation was conducted to determine the molecular mechanism of thymoquinone [63]. Thymoquinone activity in malignant glioma cells (U87MG and T98G) induced apoptosis and cell death by inhibiting proteasome activity. This inhibitory effect results in an enhancement in the level of apoptotic proteins, such as p53 and Bax, leading to the activation of the apoptosis cascade. Findings suggested the very low toxicity of thymoquinone, which is worth clinical analysis and examination mainly for its potential application as an adjuvant in cancer treatment [74]. Additionally, the inhibitory effect of thymoquinone on the NF signaling pathway and apoptosis induction was investigated and confirmed in KBM-5 leukemia cells [85]. In general, thymoquinone induced p53-dependent checkpoint kinase-1 (CHEK-1) in HCT-116 colorectal cancer cells. Since p53 mutation accounts for >50% of colorectal cancer cases and CHEK-1 activation declines the potency of DNA damaging drugs, it was speculated that thymoquinone in combination with CHEK-1 inhibitors might be effective against colorectal cancer. However, no data was provided to report whether p53 mutants are capable of suppressing CHEK-1 differently [93].

The activation of the signal transducer and the activator of the transcription 3 (STAT3) signaling pathway is an indication of tumor survival and cell proliferation [94]. It has been found that STAT3 is activated through ligands binding to inflammatory cytokines (IL-6) and growth factors and initiates a downstream signaling pathway via recruitment of adaptor kinases (JAKs and SRC) [95]. Thymoquinone has the potential to inhibit IL-6 induced STAT3 activation in multiple myeloma (MM) cells (U266 and RPMI8226), followed by blocking JAK and SRC, resulting in the suppression of the downstream signaling pathway. On the other hand, thymoquinone potentially blocked the expression of STAT3 regulatory genes, such as cyclin D1, Bcl-2, Bcl-xL, survivin, MCI-1 and VEGF [96]. It also induced cell cycle arrest at the G1 phase and activated apoptosis. Thymoquinone, in combination with thalidomide and bortezomib, enhanced the apoptotic effect of the drugs in multiple myeloma (MM) cells through the inhibition STAT3 signaling pathway [96]. Although thymoquinone was introduced as a potentially effective suppressor of proliferation and angiogenesis via inhibition of STAT3, a further in vivo study may highlight thymoquinone as a potential therapeutic agent in cancers harboring active STAT3.

A significant anti-tumor activity of thymoquinone against a 20-methylcholanthrene (MC)-induced fibrosarcoma tumor was detected through antioxidant activity and interference with DNA synthesis coupled with the enhancement of the detoxification process [97]. Barron and collaborators [98] also tested thymoquinone as an antioxidant agent in combination with selenium on osteoblast cells (MG63). This combination was successful in decreasing the cell count and elevating cellular damage via the inhibition of alkaline phosphatase (ALP) and mitochondrial glutathione, followed by caspases activation and cell death [98]. Thymoquinone, in another study, showed significant inhibition of cell proliferation with induction of apoptosis in p53-null osteosarcoma cells (MG63) [64]. Thymoquinone’s mechanism of action was through initiating caspase 9 and the activation of downstream caspase 3. This had suggested that thymoquinone induced apoptosis through a p53-independent mechanism because osteosarcoma malignancies with the loss of p53 activity are not responsive to a wide range of chemotherapeutic drugs. Thus, a synergistic study suggested that thymoquinone might be a supportive measurement against resistant p53-null osteosarcoma to conventional treatment [64]. Moreover, another study suggested the antiproliferative effect of thymoquinone in osteosarcoma SaOS-2 cells via the inhibition of the NF-_K_B pathway. This study suggested the use of thymoquinone as an adjuvant to conventional chemotherapy in clinical trials [65]. The cytotoxic effect of thymoquinone on HeLa cells was reported through a p53-dependent pathway [66]. Thymoquinone suppressed colon cancer cells proliferation (HT-29, HCT-116, DLD-1 and LoVo, Caco-2) with no inhibitory effect on normal intestinal cells (FHs74lnt). Further investigations on DLD-1 cells underlined the apoptotic cell death mechanism for the thymoquinone inhibitory effect; this effect was induced by the generation of ROS. Thymoquinone enhanced the phosphorylation of JNK and ERK (mitogen-activated protein kinases (MAPK)). These findings linked the pro-oxidant activity of thymoquinone with its apoptotic efficacy in colon cancer along with a protective role of MAPK. [67].

At the cellular level, thymoquinone was suggested to act as a pro-oxidant upon exposure to copper and lead to ROS-induced DNA breakage and cell death. The suggested mechanism for this effect was contemplated to be a non-enzymatic copper-dependent pathway for the cytotoxic effect of thymoquinone that may be able to mobilize and reduce endogenous copper [68]. The clinical application of CB1958 (a chemotherapy drug) is associated with hepatotoxicity. The application of thymoquinone in combination with CB1954 reduced hepatotoxicity and improved the anticancer effect of the chemotherapy in the resistant mouse mammary gland cell line (66cl-4-GFP). Indeed, thymoquinone inhibited angiogenesis, which resulted in hypoxia in tumor tissue and mediated the transformation of CB1954 into a therapeutic form. This explains the enhancement effect of thymoquinone in cancer cells treated with CB1954 in combination with thymoquinone [69]. In continuation of the thymoquinone anticancer effect, the amplification of antioxidant delivery also targeted prostate cancer treatment in the LNCaP cell line [70,71]. Additionally, thymoquinone via GSH depletion induced ROS generation and growth inhibition in C4-2B and PC-3 prostate cancer cells. The findings suggested an up-regulation of Gadd45α and apoptosis-inducing factor (AIF) and down-regulation of Bcl-2 family proteins as the effect of thymoquinone in prostate cancer cells [72]. Thymoquinone induced mitochondrial damage, caspase 8 and caspase 3 activation and apoptosis in p53 null HL-60 cells. Caspase 8 induced activation by thymoquinone has highlighted the impact of the death receptor system. To consider further investigations, it would be effective to examine whether the death receptor/ligand system (CD95 (APO-1)/Fas) is associated with thymoquinone-induced caspase 8 activations. It might also be worth investigating whether thymoquinone has any effect on the Fas-involving proteins with the death domain [73]. In HepG2 cells, thymoquinone also induced apoptosis by activating caspases 9 and 3 and cell cycle arrest at the G1/S phase [75].

Thymoquinone in papilloma (SP-1) cells stimulated G_0_/G_1_ arrest due to the enhancement of p16 expression (cyclin-dependent kinase inhibitor) and suppression of cyclin D1 expression [76]. A similar molecular mechanism of thymoquinone was also found in the apoptosis induction of breast cancer cells (T-47D and MDA-MB-468) [77]. In l7 carcinoma cells, thymoquinone blocked cellular growth via G_2_/M phase arrest; this mechanism is associated with elevated p53 protein expression and the down-regulation of cyclin B1 protein expression. Further studies are required to focus on examining the effect of thymoquinone on other cell-cycle and apoptosis regulators in an attempt to understand the exact mechanism of action of thymoquinone [76]. Later, the effect of thymoquinone on glioblastoma cancer cells (M059K and M059J) was revealed due to the inhibition of telomerase, which resulted in telomere attrition, DNA damage and cell death. Further research must be directed downstream of DNA-PKcs, which is necessary for external agent-induced telomere attrition in human cells [78]. Another published study revealed a cytotoxic effect of thymoquinone on Neuro-2a mouse neuroblastoma cells via the activation of caspase 3 and the down-regulation of XIAP caspase inhibitor. However, additional studies are required to picture the genome-wide effects of thymoquinone to explore its curative potential [79].

Aberrant expression of glycoprotein mucin 4 (MUC4) is a marker of pancreatic cancer cells (FG/COLO357). Thymoquinone down-regulated the expression of MUC4 via proteasomal degradation. This has activated apoptosis in pancreatic cancer cells through JNK signaling activation and the p38 mitogen-activated protein kinase pathway [80]. A pre-clinical in vivo study model must be carried out to examine the therapeutic effect of thymoquinone more precisely.

Doxorubicin administration is associated with ROS generation and cardiotoxicity. Thymoquinone as an antioxidant agent reduced cardiotoxicity in mice without disturbing the anti-tumor activity of doxorubicin. This effect was proven by the alteration of serum cardiac biomarker levels as a result of thymoquinone treatment. This study suggested that thymoquinone does not interfere with the anti-tumor activity of doxorubicin rather it functions as a powerful selective cytoprotective compound, which ameliorates cardiotoxicity without reducing doxorubicin’s anticancer effect [81]. Thymoquinone also induced protection against carcinogenesis and toxicity in mice via the induction of detoxifying enzymes, such as quinone reductase (QR) and glutathione transferase (phase 2 enzymes) [82]. Future investigations can be focused on the evaluation of thymoquinone phase 2 enzyme’s induction in various animal species using different doses, routes and longer administration periods. Thymoquinone enhanced CD8^+^T cell activity and CD62L receptor expression, which increased the expression of interferon-gamma, suggesting that thymoquinone conditioned T cells against cancer progression [83].

The successful treatment of thymoquinone against several cancer cell lines ((lung (LNM35), liver (HepG2), colon (HT29), melanoma (MDA-MB-435), breast (MDA-MB-231 and MCF-7)) were also reported to be through the activation of intrinsic apoptosis pathway upon the suppression of Akt phosphorylation. Thymoquinone also supported the cisplatin anticancer effect in tumor cells. The inhibitory effect was associated with highly expressed caspase 3. In silico target, determination suggests that thymoquinone induced DNA damage by targeting histone deacetylase activity (HDAC) and human 15-hydroxyprostaglandin dehydrogenase (HPGD) [84]. Another study showed the promising anticancer effect of thymoquinone was through its interference with polyp progression in ApcMin mice via the induction of tumor cell-specific apoptosis and controlling the Wnt signaling pathway by activating GSK-3 beta. This activation resulted in the accumulation of β-catenin and a decline in the expression of nuclear c-Myc [86]. Apoptosis activation through p38 inhibition was also detected as a result of thymoquinone treatment in T28 oral cancer cells; however, there was no animal study carried out to support this finding [87]. Thymoquinone also induced apoptosis in squamous cell carcinoma (A431, HEp-2, and RPMI 2650) via targeting caspase cascade, JNK and Akt pathways [88]. The anticancer effects of thymoquinone on human astrocytoma cells (U87) and human T lymphocyte cell line Jurkat (leukemia cell) disclosed the degradation of alpha/beta-tubulin. This resulted in the overexpression of p73 (tumor suppressor protein) followed by apoptosis induction in cancer cells. However, apoptosis was not detected in the non-cancerous human fibroblast cells suggesting the selective activity of thymoquinone on alpha/beta-tubulin in cancer cells. Since thymoquinone is a small drug-like molecule, it is more likely this compound will decline alpha/beta-tubulin level in cancer cells via the enhancement of proteasomal degradation. Although this study reported the selective degradation of alpha/beta-tubulin proteins in astrocytoma and leukemia cells, the mechanism involved in the inhibitory effect of thymoquinone has yet to be clarified [89].

**Figure 6:**
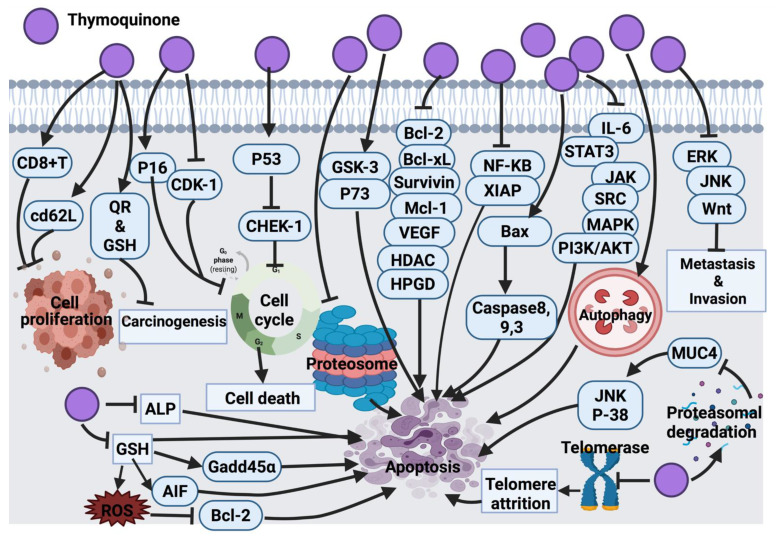
Molecular mechanisms involved in the effect of thymoquinone on cancer cells. CD8^+^T: killer T cell, cd62L: L-selectin, QR: quinone reductase, GSH: glutathione, ALP: alkaline phosphatase, AIF: apoptosis inducing factor, Bcl-2: B-cell lymphoma 2, Gadd45α: growth arrest and DNA damage inducible alpha, p53: tumor protein, CHEK-1: checkpoint kinase-1, GSK-3: glycogen synthase kinase-3, p73: tumor protein 73, Bcl-xL: B-cell lymphoma-extra large, Mcl-1: Mcl-1 apoptosis regulator, VEGF: vascular endothelial growth factor, HDAC: histone deacetylases, HPGD: 15-hydroxyprostaglandin dehydrogenase, NF-_K_B: Nuclear factor kappa light chain enhancer of activated B cells, XIAP: X-linked inhibitor of apoptosis protein, Bax: Bcl-2 associated X protein, IL-6: interleukin-6, STAT3: signal transducer and activator of transcription 3, JAK: Janus kinase, SRC: oncogene, MAPK: mitogen activated protein kinase, PI3K/Akt: Phosphoinositide 3-kinase/ Protein kinase B, ERK: extra cellular signal-regulated kinase, JNK: c-Jun N-terminal kinase, Wnt: wingless related integration site, MUC4: mucin 4, p38: mitogen activated protein kinase, ROS: reactive oxygen species.

A more recent study revealed the anticancer effect of thymoquinone via the activation of caspase 9 in glioma and prostate cancer cell lines (T98, LNCaP and 3T3) [90]. A similar anticancer mechanism of action of thymoquinone was also detected in renal carcinoma cell lines (BFTC909, 786-O and 786-O-SI3). In addition, thymoquinone suppressed the aldehyde dehydrogenase enzyme, Nanog and Oct-4 transcription factors, Nestin (metastatic marker) and CD44, which resulted in tumor growth inhibition [91]. Zhang et al. [92] indicated that thymoquinone has the potential to inhibit the metastasis of human renal cancer cells (ACHN and 786-O) via induction of autophagy through the AMPK/mTOR signaling pathway. This has suggested a combination of thymoquinone with autophagy inhibitors as a future remark to increase thymoquinone-induced anticancer activity. Figure 6 represents a summarized view of the anticancer mechanism of thymoquinone.

### 3.2. Sesquiterpenoids and Sesquiterpene Lactones

#### 3.2.1. Sesquiterpene Lactones

**Figure 7:**
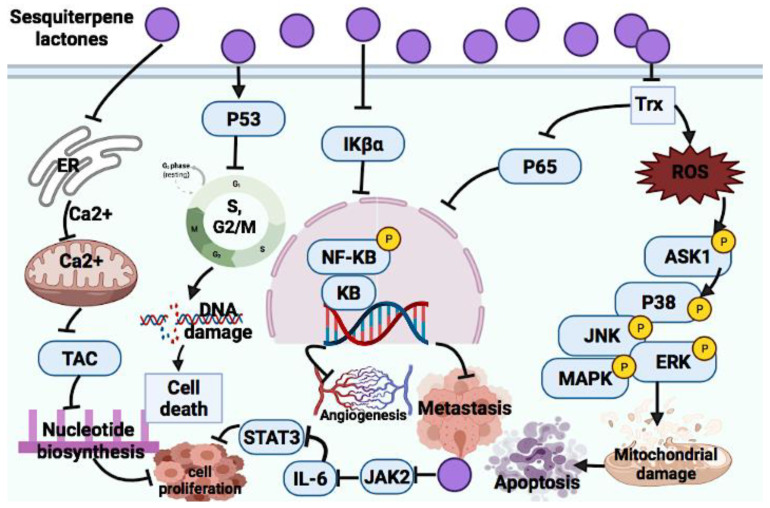
Molecular mechanisms involved in the effect of sesquiterpene lactones on cancer cells. ER: endoplasmic reticulum, Ca^2+^: calcium ion, TAC: Tricarboxylic acid, p53: tumor protein, IKβα: NF-kappa-B inhibitor alpha, NF-_K_B: Nuclear factor kappa light chain enhancer of activated B cells, KB: kappa light chain enhancer of activated B cells, P: phosphorylation, p65: oncogene, Trx: thioredoxin system, ROS: reactive oxygen species, ASK1: apoptosis signal-regulating kinase 1, p38: mitogen-activated protein kinase, JNK: c-Jun N terminal kinase, ERK: extracellular signal-regulated kinase, MAPK: mitogen-activated protein kinase.

Sesquiterpene lactones consist of huge secondary plant metabolites. This group of terpenoids, for example, ambrosin, coronopilin and dindol-01, have been reported to exert DNA damage in breast cancer cells (MCF-10A, MCF-7, JIMT-1 and HCC1937) due to cell cycle arrest at the S and G_2_ phases, which enhanced p53 protein expression [99]. An anticancer mechanism of sesquiterpene lactone in the extract of certain Mexican Indian medicinal plants was suggested to be through the inhibition of the NF-_K_B signaling pathway [210]. Sesquiterpene lactone extracted from *Artemisia macrocephala* showed anticancer effects against 3T3, HeLa and MCF-7 cells through ROS generation, targeting the sarcoplasmic reticulum calcium ATPases pump, NF-_K_B, p53 signaling pathway, angiogenesis and metastasis [211]. The significant anticancer roles of sesquiterpene lactones in oxidative stress-mediated apoptosis were marked and reported through the suppression of NF-_K_B, STAT3 and the involvement of p53 protein [212]. Here, the anticancer mechanism of action of some sesquiterpene lactones are discussed and Figure 7 illustrates the summary of mechanism of action.

##### Parthenolide

Parthenolide is another example of natural sesquiterpene lactones that exerts its anticancer effects via NF-_K_B and STAT inhibition and suppression of pro-apoptotic genes [100]. Moreover, some other studies reported the anticancer effect of parthenolide with a similar anticancer mechanism [101,102]. Abnormal activation of the STAT3 pathway is linked to cancer development. Jak2 is one of the upstream kinases of STAT3 that can be activated in response to cytokine stimulation, and it is a main kinase in the IL-6-induced STAT3 phosphorylation. Parthenolide was reported as a strong suppressor of JAK2. This compound covalently changed Cys178, Cys243, Cys335 and Cys480 of Jak2 to inhibit kinase activity [103]. In addition, ROS-mediated apoptosis is another molecular mechanism of parthenolide [104]. As a future recommendation, the cell-specific activity of parthenolide should be investigated.

##### Costunolide

Costunolide as a sesquiterpene lactone exerts its anticancer effect via targeting G2/M phase arrest in breast cancer cells (MCF-7, MDA-MB-231). It down-regulates positive cell cycle regulators, such as cyclin D1, cyclin D3, CDK4 and CDK6, while up-regulating negative cell cycle regulators, such as p18 INK4c, P21 CIP/Waf-1 and p27 KIP1. These lead to the induction of apoptosis. Costunolide was reported to activate both intrinsic and extrinsic apoptosis pathways via mitochondrial-mediated apoptosis and the activation of FasL and TNF-α protein expression [105].

##### Dehydrocostus Lactone

This sesquiterpene lactone induces apoptosis in HepG2 and PLC/PRF/5 cells by targeting the up-regulation of apoptotic proteins, such as Bax and Bak, and the down-regulation of anti-apoptotic proteins, such as Bcl-2, Bcl-XL, AIF and endonuclease G (Endo G) [106].

##### Helenalin

Helenalin was reported to induce an anticancer effect via the inhibition of human telomerase reverse transcriptase (hTERT) and telomerase in hematopoietic cancer cells [107]. It also mediated apoptosis in activated CD + T cells via mitochondrial apoptosis induction [108]. Another anticancer molecular mechanism of this compound is the inhibition of NF-_K_B by targeting P65 expression [109]. Another study revealed the anticancer effect of helenalin against A2780 human ovarian cancer cells via NF-_K_B p65 repression. In addition, several studies suggested the role of NF-_K_B p65 in autophagy-induced cell death; however, the exact mechanism in which helenalin triggers autophagy is still not clear [110].

##### Elephantopus Mollis 23 (EM23)

EM23, a sesquiterpene lactone, has the potential to suppress human chronic myeloid leukemia K562 cells and acute myeloid leukemia HL-60 cells via apoptosis induction. EM23 inhibits the expression of the thioredoxin system (Trx) and leads to the disruption of the cellular redox balance, which stimulates the apoptosis signal-regulating kinase 1 (ASK1) and its downstream factors, such as p38, JNK and ERK MAPKs, and induces apoptosis [111].

##### Artesunate and Artemisinin

Artesunate, a sesquiterpene lactone, is a strong anti-malaria drug [112]. The anticancer effect of artesunate was reported in H22 solid hepatic carcinoma cells, and the proposed mechanism of action was via the down-regulation of proliferating cell nuclear antigen (PCNA) and Bcl-2 gene expression and the up-regulation of Bax gene level [113]. Synergistic anticancer effects of artesunate in combination with allicin revealed the inhibition of osteosarcoma (MG-63 and U20S) cell growth via mitochondrial apoptosis-dependent pathway evidenced by the expression of caspase 3/9 [124]. Another study reported that artesunate mediated head and neck cancer cell’s (HNC) death via the Nrf2 antioxidant response element (ARE) pathway, which inhibited ferroptosis resistance and induced iron-dependent ROS-mediated ferroptosis. Artesunate induced ferroptosis via the declining GSH level and enhancing lipid ROS level [131]. The anti-tumor effect of artesunate in uveal melanoma cells is exerted via the inhibition of β-catenin accumulation and the activation of downstream targeted genes, such as c-Myc and CDK1 [132]. Suppression of accumulation of β-catenin was also detected in colorectal cancer as a result of artesunate treatment in an in vivo study, which caused the induction of apoptosis [133]. The anticancer effect of artesunate and its safety warrant further investigations using appropriate animal models to validate the findings and to explore the exact mechanism of action.

In HCT-116 colorectal cancer cells, artesunate induced apoptosis by up-regulating the level of proteins associated with the mitochondrial death pathway. Moreover, autophagy was also detected as a death mechanism in this cell line. It is critical for future studies to co-administer artesunate with autophagy inhibitors, which might enhance the anticancer efficacy [134]. The anticancer effect of artesunate on the same cell line was reported to be through the mitochondrial apoptosis pathway and suppression of the NF-_K_B pathway [135]. Another study suggested that the anticancer effect of artesunate on CLY colorectal cancer cells is exerted via the inhibition of the Wnt/beta-catenin pathway. Researchers suggested that with inducing apoptosis, beta-catenin is degraded by caspase 3. Thus, they assumed that a decline in beta-catenin level appeared to be a downstream effect of apoptosis. However, the activity of artesunate against cancer was restricted to cancer cells with an over-activated Wnt/beta-catenin signaling pathway [136]. Ji et al. found that artesunate-loaded nanoparticles enhanced drug uptake in cervical carcinoma cells and induced ROS-mediated apoptosis [137]. Furthermore, artesunate suppressed proliferation of breast cancer (SK-BR-3, MDA-MB-468, MCF-7 and MDA-MB-231) cells via ROS-dependent G_2_/M phase arrest and ROS-independent G_1_ arrest. The cytotoxic effect of artesunate was decreased under hypoxic conditions, which is in line with the ROS-dependent cytotoxic activity of artesunate against breast cancer cells. This research proposed that breast cancer cells under hypoxic conditions might develop resistance against the cytotoxic effect of artesunate. Therefore, hyperbaric oxygen treatment, which enhances the efficacy of ROS-dependent radiotherapy, can be considered to overcome the repressive effect of hypoxia on artesunate-induced cytotoxicity [114].

In pituitary adenoma (GH3 and MMQ) cells, artesunate exerted its inhibitory effect in combination with bromocriptine (BRC) by suppressing miR-200c expression and enhancing PTEN expression. This combination could be further explored in future clinical trials as a potential strategy to enhance the therapeutic efficacy against prolactinoma [115].

Artesunate in combination with carboplatin exerted synergistic anticancer effect against lung cancer cells via activating mitochondrial-dependent apoptosis through G_2_/M phase arrest and the up-regulation of Bax, p21, p53 and caspase 3 expression [116]. Another study revealed the anticancer effect of artesunate on A549 lung cancer cells in in vitro and xenograft study models where the inhibitory effect of this compound involved the down-regulation of EGFR, Akt and ATP binding cassette subfamily G member 2 (ABCG2). It was hypothesized that artesunate at low concentrations did not suppress ABCG2 activity, but high concentrations may exert an inhibitory effect. Thus, this claim can be further tested in future studies [117]. Artesunate induced apoptosis in TE671, RD18 and C2C12 myoblast cells via ROS generation and inhibition of the p38 MAPK pathway. Indeed, artesunate treatment in myoblast cells induces myo-miRs (miR-133a and miR-206) expression, which, in turn, reduces PAX7 protein (a transcription factor) expression. Eventually, artesunate overexpresses adhesion molecules, such as NCAM and integrin β1, followed by a decline in the in vitro migration and invasiveness of myoblast cells. Moreover, artesunate reduced myoblast tumor growth in xenograft animal models by approximately 50% [118]. However, artesunate might act differently towards various tumors; therefore, an extensive investigation is required to explore the potential use of artesunate as a therapeutic agent.

Mitochondrial apoptosis was also detected as an underlying mechanism of action of artesunate in retinoblastoma (WERI-Rb1) cells and Sprague-Dawley rats through the up-regulation of Kruppel-like factor 6 (KLF6). Since artesunate is a safe therapeutic agent and showed high efficacy against retinoblast cells, future clinical trials are recommended [119].

Heat shock proteins are a target for tumor treatment. Artesunate in combination with triptolide in in vitro and in vivo studies declined the expression of heat shock proteins (HSP20 and HSP27) and blocked proliferation pathways related to these proteins in pancreatic cells (PANC-1 and CFPAC-1) [120]. In another study, synergistic anti-tumor effects of artesunate and sorafenib were detected against hepatocellular carcinoma cells (PLC/PRF/5, HuH7, HepG2, Hep3B and HCCLM3) through targeting the ERK pathway and STAT3 [121]. Artesunate disrupted tyrosine phosphorylation of STAT-3 in HepG2 hepatocellular carcinoma cells [122]. Artesunate was also found to have an anti-metastatic effect in cervical cancer in an in vivo study model via the suppression of HOX antisense intergenic RNA (HOTAIR) expression, which, in turn, declined COX-2 expression at the post-transcriptional level. However, whether other molecules are associated with the interaction of HOTAIR as oncogenic non-coding RNA and COX-2 needs further investigations [123].

Artesunate triggers esophageal cancer cell (Eca109) apoptosis and cell cycle arrest via targeting mitochondrial membrane potential, Bcl-2, Bax caspase 3 and CDC25A expression levels, leading to G1/S transition impairment and prevention of cell growth. Knowing that artesunate is an approved anti-malaria drug with minimal side effects could attract scientists’ attention towards artesunate repurposing against esophageal cancer and conducting future clinical studies [125]. Artesunate suppressed phosphorylation of certain proteins involved in cell survival (p38, ERK, STAT5, CREB) and induced expression of SOCS-1 protein and apoptosis via caspase 3 activation in chronic myeloid leukemia (CML) xenograft mouse model. Further analysis showed the suppression of certain anti-apoptotic genes (Bcl-2, Bcl-xl, survivin) were inhibited by artesunate. These findings suggest the inhibitory effect of artesunate on transcriptional factors and induction of apoptosis [126].

**Figure 8:**
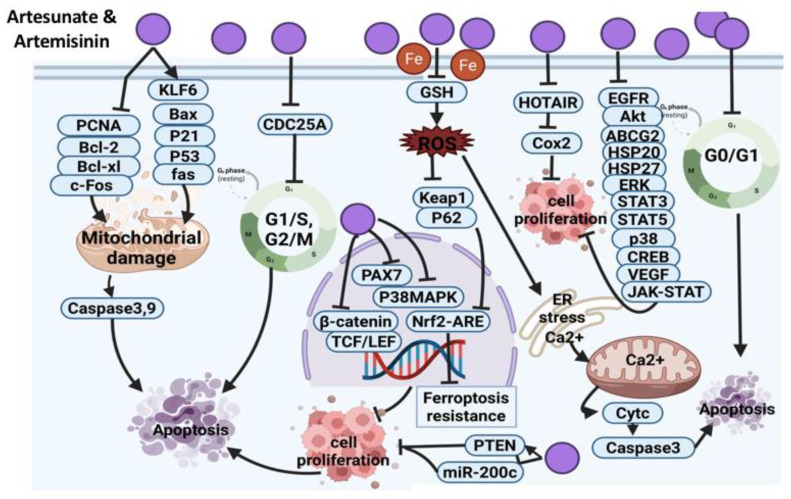
Molecular mechanisms involved in the effect of artesunate and artemisinin on cancer cells. PCNA: proliferating cell nuclear antigen, Bcl-2: B-cell lymphoma 2, Bcl-xL: B-cell lymphoma-extra large, c-Fos: Fos proto-oncogene, KLF6: Kruppel like factor 6, Bax: Bcl-2 associated X protein, p53: tumor protein, fas: cell surface death receptor, CDC25A: cell division cycle 25 A, GSH: glutathione, ROS: reactive oxygen species, Keap1: Kelch-like ECH-associated protein 1, p62: ubiquitination protein, PAX7: paired box 7, p38 MAPK: mitogen activated protein kinase, Nrf2: nuclear factor erythroid 2-related factor 2, ARE: antioxidant response element, TCF/LEF: T cell factor/lymphoid enhancer factor family, PTEN: phosphatase and tensin homolog, HOTAIR: HOX transcript antisense RNA, Cox2: cyclooxygenase 2, ER: endoplasmic reticulum, Ca^2+^: calcium ion, EGFR: epidermal growth factor receptor, Akt: Protein kinase B, ABCG2: ATP binding cassette subfamily G, HSP20: heat shock protein 20, HSP27: heat shock protein 27, STAT3: signal transducer and activator of transcription 3, STAT5: signal transducer and activator of transcription 5, CREB: cAMP-response element binding protein, VEGF: vascular endothelial growth factor, JAK-STAT: Janus tyrosine kinase-signal transducer and activator of transcription, Cyt c: cytochrome *C*.

Artesunate was also tested against gastric cancer cells (SGC-7901, BGC-823 and AGS) for its anticancer activity. As a result, artesunate inhibited cell growth in a concentration-dependent manner (0–260 μM/L). Artesunate treatment in BGC-823 cells showed features of necrosis rather than apoptosis. The anticancer mechanism of artesunate against BGC-823 cells involved the suppression of VEGF protein, a key initiator of tumor angiogenesis. This suppression was accompanied by an elevated Ca^2+^ level in the cells. This has suggested the down-regulation of VEGF protein association in the artesunate-induced oncosis in BGC-823 cells [127]. Additional studies are essential to understand the molecular mechanism of oncosis as a complex network composed of many factors. The potential anticancer effect of artesunate against human epidermoid carcinoma cells (A431), HepG2 hepatocellular carcinoma and HaCaT spontaneously immortalized human keratinocytes cells was suggested to be through G_0_/G_1_ cell cycle arrest and iron-dependent mitochondrial apoptosis [128]. Artesunate supplementation to nitrosodiethylamine-administered animals declined liver proliferation-associated tumorigenesis through JAK-STAT signaling inhibition [129]. In the K562 cell line and U937 human leukemia cells, artesunate induced an intrinsic apoptosis pathway by enhancing Fas expression and inhibiting c-Fos expression level [130]. The overall anticancer molecular mechanism of artesunate is summarized in Figure 8.

##### 3.2.2. β-Elemene

**Figure 9:**
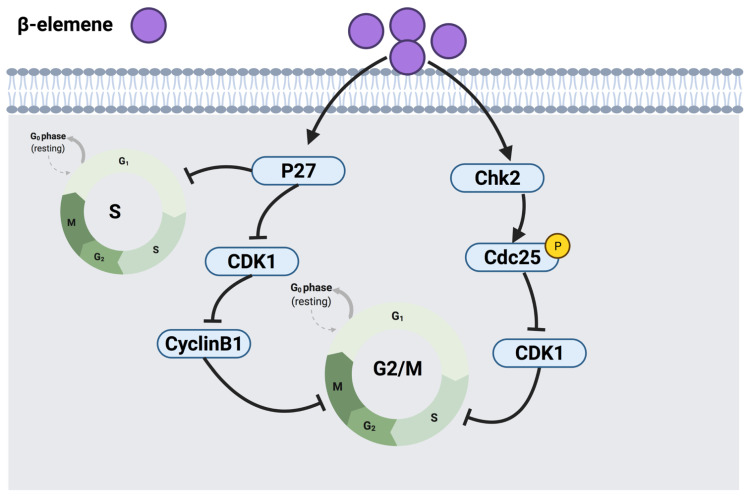
Molecular mechanisms involved in the effect of β-elemene on cancer cells. p27: cyclin-dependent kinase inhibitor 1B, CDK1: cyclin-dependent kinase 1, Chk2: checkpoint kinase 2, CDC25: cell division cycle 25, P: phosphorylation.

β-elemene, another sesquiterpenoid, is one of the active constituents of the Chinese herbal medicine *Curcuma wenyujin*. This compound has been investigated in human NSCCL cell lines (H460 and A549). Findings revealed that β-elemene arrests cell cycle at G2/M and S phases. Indeed, β-elemene exerted its mechanism of action by up-regulating p27 as an inhibitor of CDK1, hence reducing phosphorylation of CDK1 on threonine and decreasing the level of cyclin B1 expression, leading to under-expression of cyclin B1-CDK1 multiplex and G2/M phase cell cycle arrest [138]. In addition, β-elemene was demonstrated to up-regulate the expression of a checkpoint kinase (Chk2), resulting in the phosphorylation of CDC25C followed by the down-regulation of CDK1, thus arresting cells at the G2/M phase through a Chk2-dependent mechanism [138]. In addition, β-elemene had an inhibitory effect on the expression level of cyclin A in human lung cancer cells and disrupted the formation of the cyclin A-CDK2 complex. On the other hand, CDK2 requires phosphorylation on threonine 160 for its activation, which was inhibited by the up-regulated p27 upon exposure to β-elemene and resulted in S phase arrest and apoptosis. Additional studies are warranted to determine the exact mechanism of β-elemene in terms of how it affects the expression level of cyclin-A, cyclin B1, CDC25C, Bcl-2 genes, and whether β-elemene-induced cell cycle arrest is linked to apoptosis [139]. Figure 9 illustrates a summarized graph of the anticancer molecular mechanism of β-elemene against cancer cells.

### 3.3. Diterpenoids

#### 3.3.1. Triptolide

Triptolide at low concentrations (1 ng/mL) induced senescence-like phenotype in tumor-derived human prostate epithelial cells. On the other hand, at higher concentrations (50–100 ng/mL), triptolide induced apoptosis, where the p53 protein level increased in response to triptolide treatment [140]. The molecular signaling pathway underlying the two modes of cell death by low and high concentrations of triptolide required further research. Another study showed that the progression of prostate cancer in the xenograft model was inhibited by triptolide via limited SUMO-specific protease 1 activity (SENP1), as well as androgen receptor expression and c-Jun transcription activity. Additional studies are necessary to confirm whether triptolide down-regulates SENP1, c-Jun and androgen receptors via targeting XPB and Rpb1 (targets of triptolide) [141].

One of the main molecular targets of triptolide is the X-box binding protein 1 (XBP1) subunit of transcription factor II H (TFIIH) [147]. Triptolide, upon its binding with XBP1, suppresses the transcription process and polymerase II activity [147,148]. Furthermore, triptolide binds to xeroderma pigmentosum type B (XPB), a part of the TFIIH complex, and results in the degradation of Rpb1. This binding, via an unknown mechanism, activates Cdk7 and p44, which triggers Ser-5 phosphorylation of RNA polymerase II subunit Rpb1. This may inhibit RNA polymerase II and ease poly-ubiquitination induced by the activated p44. Eventually, this poly-ubiquitination leads to proteasome-dependent degradation. However, the ubiquitination site in Rpb1 in response to triptolide was not determined; therefore, it cannot be excluded that Rpb1 is ubiquitinated outside of the carboxy-terminal domain [149]. In human epidermoid carcinoma cells (KB, KB-7D and KB-tax), triptolide induced cell growth inhibition via apoptosis [150]. The inhibitory mechanism of triptolide occurs by the suppression of RNA polymerase II in a CDK-dependent pathway. Moreover, triptolide is capable of inhibiting P-gp, and the drug efflux results in cancer cell death [151].

Triptolide exerts anticancer effects by inhibiting focal adhesion kinase (FAK) expression, resulting in the impairment of ERK1/2 downstream signaling pathway in lung cancer cells (H460, A549 and H358) [152]. Since there are differences in the p53 status of the tested cell lines, it is worth specifically investigating the effect of triptolide treatment on p53 mutation status. In non-small-cell lung cancer cells (H1299 and NCI-H460), triptolide induced apoptosis via the inhibition of Akt, mTOR and P70S6K phosphorylation and the downstream signaling pathway. It also inhibits glycolysis via impairment of glucose utilization, HKII, glutathione (GSH) and Nrf2 activity. However, Akt inhibition might not be enough to explain glycolysis and HKII down-regulation [153]. The in vitro and in vivo inhibitory effects of triptolide against breast cancer (MCF-7 and MDA-MB-231) were reported through the inhibition of high motility group box 1 (HMGB1) [154]. Therefore, future studies should be focused on the mechanism through which triptolide modifies the expression and secretion of HMGB1. In another study, the anti-tumor effect of triptolide involved the inhibition of MDM2 protein in breast cancer cells, which triggered inhibition of Akt activation; however, future studies are necessary to explore the structure–activity relationship of triptolide [142]. The molecular mechanism of triptolide in cervical cancer cells (SiHa) was mainly through apoptosis activation by autophagy induction via the suppression of the PI3K/Akt/mTOR signaling pathway [143].

**Figure 10:**
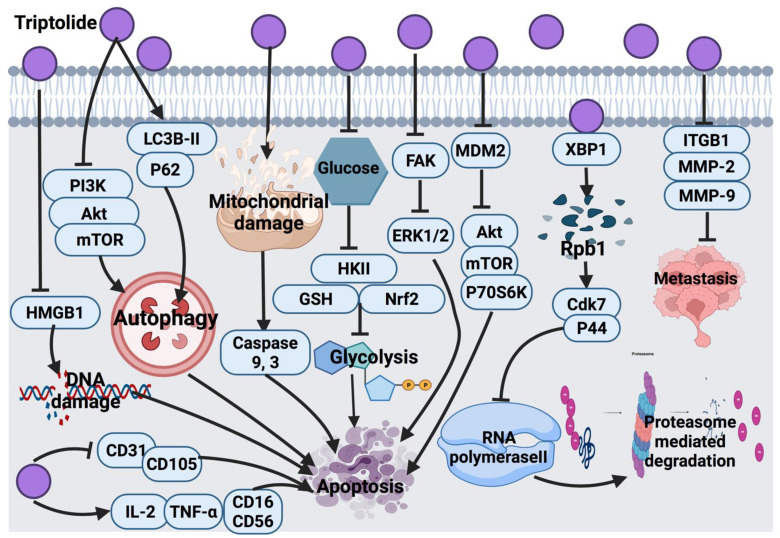
Molecular mechanisms involved in the effect of triptolide on cancer cells. HMGB1: high mobility group box 1 protein, PI3K: phosphatidylinositol 3-kinase, Akt: protein kinase B, mTOR: mammalian target of rapamycin, CD31: platelet endothelial cell adhesion molecule, CD105: endoglin, IL-2: interleukin-2, TNF-α: tumor necrosis factor-alpha, CD16: transmembrane receptor, CD56: neural cell adhesion molecule, LC3B-II: microtubule-associated protein, p62: ubiquitin-binding protein, HKII: hexokinase 2, GSH: glutathione, Nrf2: nuclear factor erythroid 2-related factor 2, FAK: focal adhesion kinase, ERK1/2: extracellular signal-regulated kinase 1/2, MDM2: mouse double minute 2 homolog, P70S6K: ribosomal protein S6 kinase beta-1, XBP1: X-box binding protein 1, Rpb1: DNA-directed RNA polymerase II subunit, CDK7: cyclin-dependent kinase 7, p44: mitogen-activated protein kinase, ITGB1: integrin beta 1, MMP-2: Matrix metalloproteinase-2, MMP-9: Matrix metalloproteinase-9.

Triptolide exerts cytotoxic effects against MDA-MB-231 triple-negative breast cancer cells via the activation of caspase 3 and apoptosis pathway, as well as through modulation of the autophagy signaling pathway via alteration of the level of LC3B-II autophagy protein and p62 receptor, resulting in cell death [144]. Further in vitro and animal-based studies are recommended to elucidate and validate the effect of triptolide on various pathways in triple-negative breast cancer cells. In another study, triptolide suppressed metastasis of SKOV/DDP cells via reduction of the expression of adhesion-related proteins integrin beta 1 (ITGB1) and inhibiting apoptosis agents, such as MMP-2 and MMP-9 proteins [145]. An in vivo study revealed that triptolide potentially enhanced IL-2 and TNF-α expression, which elevated NK cell-related proteins, such as CD16 and CD56, and reduced the expression of proteins that promote the growth of blood vessels, such as CD31 and CD105. The authors suggested synergistic anti-tumor activity of triptolide in combination with cisplatin against epithelial ovarian cancer cells (EOC) [146]. Figure 10 shows the overall anticancer mechanism of triptolide.

#### 3.3.2. Crocetin

The anticancer effect of crocetin in human esophageal squamous carcinoma (KYSE-150) cells was confirmed via the growth inhibitory effect by targeting PI3K/AKT and ERK1/2 signaling pathways, p38 down-regulation and the overexpression of p53 and p21 proteins. These alterations mediated the mitochondrial apoptosis pathway via disrupting mitochondrial membrane potential leading to overexpression of Bax protein, caspase 3 activations and the down-regulation of Bcl-2 expression [155]. However, it is worth exploring the anticancer effect of crocetin against other cancer cell lines and validating the targets using inhibitors to confirm the link between crocetin and pathways. Crocetin combined with cisplatin exerted a synergistic effect against KYSE-150 cells via a similar mechanism of action and enhanced cisplatin apoptosis activity [156]. Another study also reported that crocetin arrests KYSE-150 cells at the S phase, alters cell morphology and enhances the expression of Bax, and caspases apoptotic genes [158].

The anticancer molecular mechanism of crocetin in primary APL cells, HL60 cells and NB4 cells via the inhibition of pro-survival genes, such as Akt and Bcl-2, multidrug-resistant proteins (ABCB1 and ABCC1) and tyrosyl-DNA phosphodiesterase 1 (TDP1), as well as the overexpression of pro-apoptotic genes, such as caspase 3, 9 and Bax [159]. The tumor preventive effects of crocetin on N-methyl-N nitrosourea (NMU)-induced breast cancer in rats was also examined, where the inhibitory molecular mechanism triggered alterations in Bax and Bcl-2 expression levels, caspase activation, cell cycle arrest via cyclin D1, p21, and p53 modulation and interaction with DNA sequence [160].

**Figure 11:**
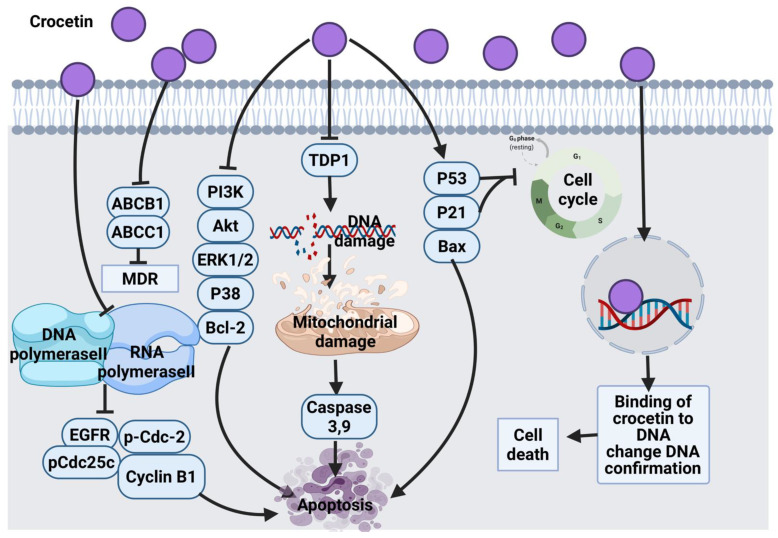
Molecular mechanisms involved in the effect of crocetin on cancer cells. ABCB1 and ABCC1: ATP binding cassette subfamily B member 1, MDR: multidrug-resistant, EGFR: epidermal growth factor receptor, pCdc25c: phosphorylated cell division cycle 25 c, p-Cdc-2: cell division control-2, PI3K: phosphatidylinositol 3-kinase, Akt: protein kinase B, ERK1/2: extracellular signal-regulated kinase 1/2, p38: mitogen-activated protein kinase, p21: cyclin-dependent kinase inhibitor 1, Bcl-2: B-cell lymphoma 2, TDP1: tyrosyl-DNA phosphodiesterase 1, p53: tumor protein, Bax: Bcl-2 associated X protein.

In AGS human gastric adenocarcinoma cells, crocetin treatment induced accumulation of cells in sub G_1_ phase, mitochondrial apoptosis and caspases signaling activation, Bax up-regulation and Bcl-2 down-regulation [161]. Minor groove binding ligands are highly targeted with anti-tumor drugs. Crocetin directly interacts with DNA minor grooves and induces alterations in the DNA conformation. The interaction of crocetin with DNA was considered as a possible anticancer mechanism in a fluorometric measurement study [162,163]. The use of sodium chloride to enhance the solubility of crocetin is recommended because the ionic strength increases the interaction of crocetin with DNA. Another study showed that crocetin exhibits a chemopreventive effect against tobacco-specific carcinogen benzo (a) pyrene-induced lung carcinogenesis in mice. This activity was reported due to the protection of glycoproteins levels caused by crocetin in serum and tissues of the mice. This could be due to the repressive effect of crocetin on polyamine synthesis and glycoproteins alteration, antiproliferative and free radical scavenging activity [164]. However, more studies should be carried out to understand the cellular and molecular response to crocetin treatment against lung cancer. Another animal study found the anticancer activity of crocetin due to its potential to scavenge free radicals in lung cancer tissue [165]. In p53 null SKOV3 ovarian carcinoma cells, HeLa cells and A549 cells, crocetin induced the expression of p21, which suppressed CDKs through a p53-independent mechanism and resulted in G_1_ phase arrest. In HeLa cells, it was also suggested that crocetin exhibits its anticancer effect through p53-dependent and independent mechanisms [157]. Although the exact protective mechanism of crocetin against cancer is not clear, it was proposed that crocetin suppresses DNA and RNA polymerase II function followed by inhibiting proliferation via impairing EGFR, p-Cdc-2, p-Cdc25c, cyclin B1 and induces apoptosis by altering Bax and Bcl-2 expression levels [213]. The solubility and bioavailability of crocetin warrant further optimization to enhance its effectiveness as an anticancer agent. Figure 11 summarizes the overall anticancer mechanism of crocetin.

#### 3.3.3. Phytol

Phytol induced an intrinsic apoptosis pathway by mitochondrial membrane depolarization in A549 lung cancer cells. The molecular mechanism of phytol in the A549 cell line was evidenced through bleb formation upon mitochondrial membrane damage and cell accumulation in the sub G_0_ phase, inhibition of Bcl-2 protein expression and induction of Bax protein expression leading to caspase 9 and 3 activations and apoptosis [166]. In AGC human gastric adenocarcinoma, phytol induced apoptosis, which was evidenced by accumulated cell population in the sub-G1 phase, down-regulation of Bcl-2, overexpression of Bax and eventually the activation of caspase 9 and 3, and PARP cleavage through mitochondrial depolarization. In addition to the mentioned pathways, phytol triggered autophagy by underlying accumulated acidic vesicle, transforming microtubule-associated protein LC3-I to LC3-II and inhibiting Akt, mTOR and p70S6K phosphorylation. The same study showed that phytol-induced autophagy can be inhibited when ROS generation is suppressed by N-acetyl-L-cysteine (ROS scavenger) [167]. Therefore, an additional investigation is required to detect the responsible markers for ROS generation after exposure to phytol; this will help to detect the exact interaction between ROS and autophagy.

In addition, the cytotoxic effect of phytol was investigated on eight cancerous cell lines (MCF-7, MDA-MB-231, HeLa, PC-3, HT-29, A-549, Hs294T, and MRC-5). In this study, phytol showed the highest activity against MCF-7 cells and the least activity on PC-3 cells. The in vitro cytotoxic molecular mechanism was not reported [214]. Similarly, another study suggested the anticancer effect of phytol via cytotoxicity induction in MCF-7 cells [168]. As a future recommendation, research on the pharmacokinetic profile of phytol is necessary to confirm the potency and anticancer efficacy of this terpenoid.

### 3.4. Triterpenoids

#### 3.4.1. Ursolic Acid

Several studies revealed the modulatory effect of ursolic acid on the expression and function of enzymes involved in mitochondria, growth inhibition and apoptosis in different in vitro cell lines (HaCaT, M4Beu, HuH7) and in vivo experimental cancer models [169,170,173,174,175]. The hepatoprotective effect of ursolic acid was investigated in a mouse model. Ursolic acid treatment in mice protected liver mitochondria against mitochondrial damage induced by the release of Ca^2+^ from mitochondrial matrix, cytochrome *C* and apoptosis induction. This suggests a direct inhibitory effect of ursolic acid on mitochondrial permeability transition, which inhibits cell growth via either caspase-dependent or independent mechanisms. It was suggested that a mitochondrial permeability transition pore is a noticeable future target in the search for the exact mechanism of the hepatoprotective effect of ursolic acid [173].

Lewinska et al. [176] reported the down-regulation of the Akt signaling pathway in breast cancer cells with different receptor statuses after exposure to ursolic acid. The suppression of Akt triggered glycolysis disruption and declined hexokinase2 (HK2), pyruvate kinase M2, ATP and lactate levels. Ursolic acid also induced cytotoxic growth inhibition in the bladder cancer cell line via increasing ROS production followed by mitochondrial damage and cell cycle arrest [177]. However, the cellular inhibitory mechanism of action of ursolic acid against cancer needs to be elucidated, and these results may encourage further investigation on the involvement of microtubules as a target for this novel, less toxic and effective anticancer compound.

**Figure 12:**
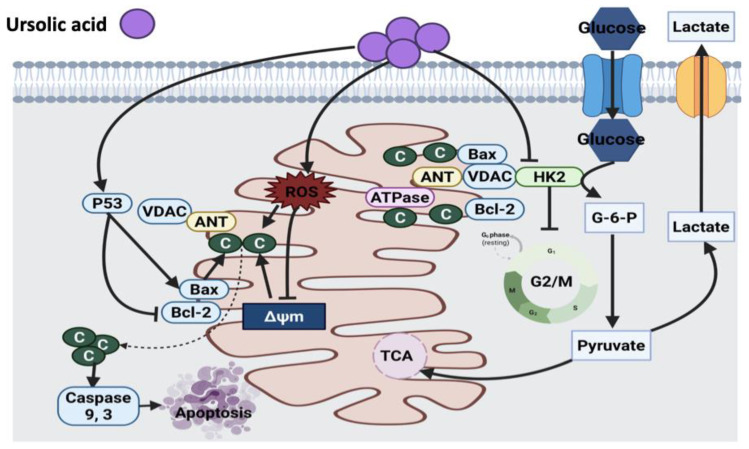
Molecular mechanisms involved in the effect of ursolic acid on cancer cells. p53: tumor protein, Bax: Bcl-2 associated X protein, Bcl-xL: B-cell lymphoma-extra large, C: cytochrome *C*, ROS: reactive oxygen species, ΔΨm: mitochondrial membrane potential, VDAC: voltage-dependent anion channel, HK2: hexokinase 2, G-6-P: glucose 6 phosphate, TCA: tricarboxylic acid.

Moreover, ursolic acid induced apoptosis in osteosarcoma cells (U-2OS and MG-63) by enhancing oxidative stress and the induction of autophagy [178]. In MDA-MB-231 cells, ursolic acid also caused cell death mainly through extrinsic apoptosis pathway via activation of Fas receptor, caspase 8 and polymerase (PARP) cleavage, up-regulation of Bax, down-regulation of Bcl-2 and release of cytochrome *C*. Moreover, ursolic acid also induced cleavage of caspase 9, thus this compound activates both intrinsic and extrinsic apoptosis pathways [179]. One shortcoming of this study is that only one breast cancer cell line was tested. In addition, the xenograft model of breast cancer could be considered to investigate the efficacy of ursolic acid for future research. Another study reported the inhibitory effect of ursolic acid on colon adenocarcinoma (SW480) cell growth via the up-regulation of p53, Bax and p21 proteins, resulting in the induction of caspase 3-dependent apoptosis [180]. A similar mechanism of action of ursolic acid was also suggested in B16F-10 melanoma cells [171] and human hepatoma cell line (SMMC-7721) [172] treated with ursolic acid. In SMMC-7721 cells, the up-regulation of p53 and Bax pro-apoptotic proteins and down-regulation of Bcl-2 anti-apoptotic protein were reported. Furthermore, an elevation in the mRNA levels of growth differentiation factor 15, superoxide dismutase 2 (SOD2) and activating transcription factor 3 were detected as well; however, p53 blocked the activity of these factors [172]. Clinical trials were suggested to fully investigate the efficacy, safety and pharmacokinetics of ursolic acid. Figure 12 illustrates the anticancer mechanism of ursolic acid.

#### 3.4.2. Betulinic Acid

Several studies demonstrated that betulinic acid induces apoptosis in cancer cells via a direct effect on mitochondria. Betulinic acid exerts the anticancer activity by inducing MMP to release cytochrome *C* from mitochondria into cytosol, leading to caspase cleavage, initiation of mitochondrial apoptosis pathway and nuclear degradation [181,182,189,190]. Other reported mechanisms of action of betulinic acid include ROS generation, p38 and MAPKs activation, which cause apoptosis in UISO-Mel-1 human melanoma cells. The study suggested conducting clinical studies to explore the clinical outcomes of betulinic acid [191].

Betulinic acid revealed a cytotoxic effect against nine human cancer cell lines (A2780, OVCAR-5, IGROV-1, H4360, A431, Me665/2/21, Me665/2/60, POGB and POGB/DX); however, the molecular mechanism of betulinic acid in this study was not investigated [192]. Betulinic acid against CD-95 resistant and CD-95 sensitive melanoma cells induced Bax/Bcl-2-independent cytochrome *C* release and DNA fragmentation [193]. Another study revealed that betulinic acid acts as an enhancer of p53 expression level in C18161 human metastatic melanoma cells [194]. The potential anticancer effect of betulinic acid via triggering apoptosis pathway was reported in several cancer cell lines (HeLa, HepG2, A549, MCF-7, NCl-H460, PC-3, SK-HEP-1 and K562) evidenced by morphological changes of cancer cells and apoptotic bodies formation [195]. Likewise, betulinic acid suppressed the proliferation of HeLa cells along with morphological changes mediated by caspase 3 up-regulation and apoptosis initiation [196].

**Figure 13:**
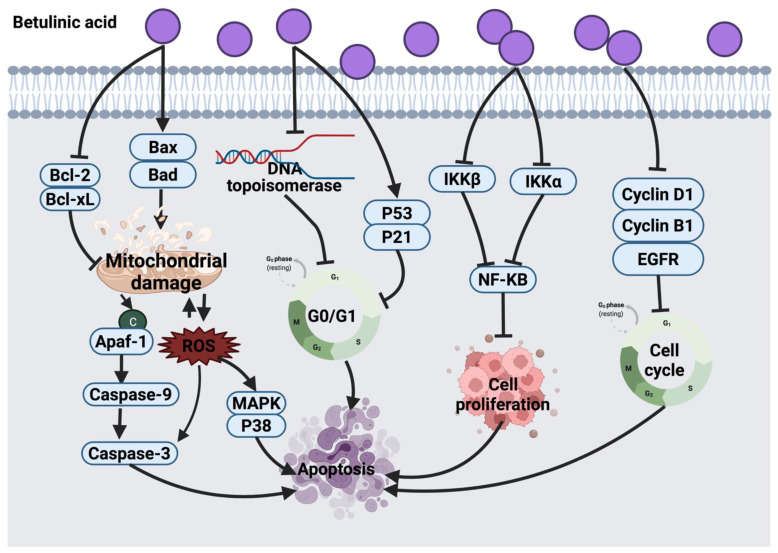
Molecular mechanisms involved in the effect of betulinic acid on cancer cells. Bcl-2: B-cell lymphoma 2, Bcl-xL: B-cell lymphoma-extra-large, Bax: Bcl-2 associated X protein, Bad: Bcl-2 associated agonist of cell death, Apaf-1: apoptotic protease activating factor 1, ROS: reactive oxygen species, MAPK: mitogen-activated protein kinase, p53: tumor protein, p21: cyclin-dependent kinase inhibitor 1, IKKα & IKKβ: core element of NF-_K_B cascade, NF-_K_B: nuclear factor light chain enhancer of activated B cells, EGFR: epidermal growth factor receptor.

In lymphoma cell lines (CL-1 and CLBL-1) and canine osteosarcoma (D-17) cells, betulinic acid treatment arrested cells in the G_0_/G_1_ phase. The anticancer effect of betulinic acid was suggested to be through suppression of topoisomerase expression [183]. Similar cell cycle arrest was also detected in HeLa cells treated with betulinic acid-rich fraction from *Dillenia suffruticosa* [184]. In breast cancer cell lines (MCF-7, MDA-MB-231, MDA-MB-453, BT474 and T47D), betulinic acid targets the suppression of cyclin and topoisomerase expression, as well as cell proliferation. These events underlined cell cycle arrest and apoptosis initiation via mitochondrial pathway initiation and inhibition of NF-_K_B, VEGF and VEGR proteins expression. However, the main target of betulinic acid in cancer cells is the estrogen receptor and multidrug-resistant proteins [185]. Betulinic acid is also expected to target cell cycle arrest at the G_0_/G_1_ phase via enhancing p53 and p21 expression levels [215] or targeting other signaling pathways, such as the mitochondrial death pathway, PI3K/Akt [184] and NF-_K_B inflammatory pathways [216]. A recent study showed that betulinic acid induced lung carcinoma (H460) cell death via G2/M phase arrest and mitochondrial apoptosis [188]. Betulinic acid can be used in combination with other chemotherapeutic agents or with TNF-related apoptosis-inducing ligand (TRAIL) molecules to increase the anticancer activity. Nevertheless, the limited water solubility of betulinic acid remains a drawback. Thus, enhancing water solubility, absorption and bioavailability of this compound is necessary before proceeding to animal and clinical trials. Figure 13 suggests a summarized view of the anticancer mechanism of betulinic acid.

#### 3.4.3. Lupeol

Lupeol is a pentacyclic triterpenoid found in edible vegetables, fruits and medicinal plants, such as grapes, cabbages and olives. Pitchai et al. [198] reported mitochondrial-mediated apoptosis in MCF-7 breast cancer cells treated with lupeol, as evident by the down-regulation of anti-apoptotic proteins. Lupeol also decreased mRNA expression of anti-apoptotic proteins and resulted in growth inhibition of lung adenocarcinoma (A549) cells [199]. Several studies have shown that lupeol exerts antiproliferative activities against prostate cancer (PC3) cells [200] and gallbladder cancer (GBC-SD) cells [201] through cell cycle arrest and via targeting PI3K and EGFR/MMP-9 signaling pathways. In addition, lupeol also inhibited melanoma (Mel-928, Mel-1241, Mel-1011) cells growth through interaction with the Wnt/β-catenin pathway via blocking the Wnt signaling pathway [60]. The growth of colorectal cancer cells (SW480 and HCT-116) was also inhibited by lupeol via cell cycle arrest at the S phase as a result of Wnt/β-catenin signaling pathway inhibition. Findings of that study showed that lupeol decreases CLDN1 in HCT-116 cells and CCNA2 in SW480 cells at both gene and protein levels. Although CCNA2 is an oncogenic gene that controls the development of colorectal cancer, cell migration and cell cycle, reduction in its expression cannot entirely describe the cell invasion and migration. Hence, further investigations to fully understand the underlying mechanism of action of lupeol are required [202].

**Figure 14:**
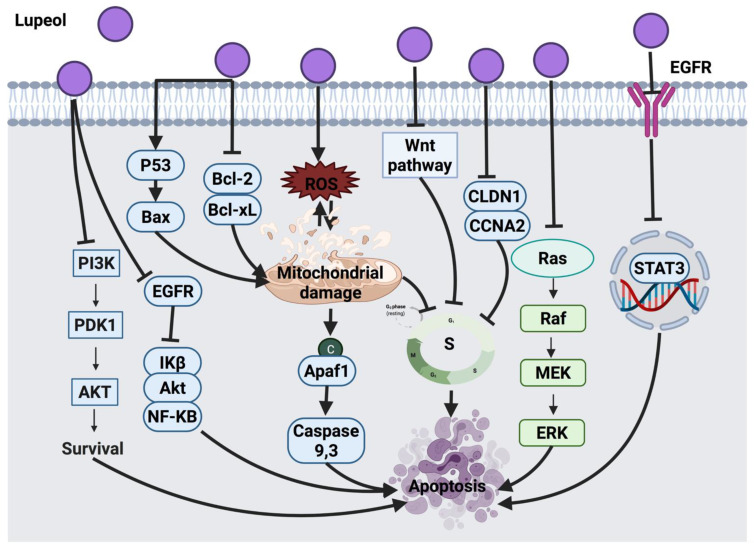
Molecular mechanisms involved in the effect of lupeol on cancer cells. PI3K: Phosphatidylinositol 3-kinase, PDK1: pyruvate dehydrogenase kinase 1, Akt: Protein kinase B, p53: tumor protein, Bax: Bcl-2 associated X protein, Bcl-2: B-cell lymphoma 2, EGFR: epidermal growth factor receptor, NF-_K_B: nuclear factor light chain enhancer of activated B cells, ROS: reactive oxygen species, Apaf1: apoptotic protease activating factor 1, Wnt: wingless-related integration site, CLDN1: claudin 1, CCNA2: cyclin A2, Ras: rat sarcoma, Raf: rapidly accelerated fibrosarcoma, MEK: mitogen-activated protein kinase, ERK: extracellular signal-regulated kinase, EGFR: epidermal growth factor receptor, STAT3: signal transducer and activator of transcription 3.

Moreover, lupeol induced apoptosis in human pancreatic adenocarcinoma cells (AsPC-1) by blocking the Ras signaling pathway, and modulating protein expression involved in PI3k/Akt, MAPKs pathways and PKC alpha/ODC [203]. However, an animal study model is required to validate these findings from a different angle. The inhibitory effect of lupeol in oral cancer cells (UPCI:SCC131 and UPCI:SCC084) was evidenced by the suppression of EGFR phosphorylation and its downstream proteins, such as protein kinase B (Akt), I kappa B (I_k_ B) and NF-_k_ B. Lupeol was further investigated in patient-derived tumor tissues. As a result, lupeol treatment declined the tumor proliferation protein Ki-67 [204]. Likewise, lupeol induced apoptosis by inhibiting EGFR phosphorylation and dephosphorylation of its downstream molecule, STAT3, in lung cancer cells (H1299, A549 and H460) [205]. However, supplementary preclinical and clinical studies were suggested to examine the anticancer effect of lupeol in depth. The anticancer effect of lupeol on cervical cancer cells (HeLa and SiHa) showed cell cycle arrest at the S phase, and mitochondrial-mediated apoptosis stimulated via superoxide generation [61]. In an in vivo xenograft model, lupeol suppressed growth of cholangiocarcinoma (CCA) tumor, and the inhibitory mechanism of lupeol was suggested via the suppression of TNF-α and downstream effectors of VEGFR-2 signaling [197]. However, the exact mechanism of pro-angiogenic and anti-angiogenic inflammatory cytokines need to be investigated. Figure 14 explains the anticancer mechanism of lupeol.

## 4. Sensitization of Cancer Cells to Chemotherapy by Certain Terpenoids

According to the literature, only some terpenoids have been reported to exhibit a sensitization effect on cancer cells to chemotherapy. A study by Bin Xu et al. reported that when MCF-7 breast cancer cells were treated with doxorubicin (DOX) in combination with β-elemene, the DOX-associated mean fluorescence intensity was elevated in cells. This indicated accumulation of DOX in resistant cancer cells upon exposure to β-elemene [217]. ATP-binding cassette (ABC) transporter activity is associated with multidrug resistance (MDR) development. β-elemene has the potential to exert its supportive effect via enhancing the fluorescent intensity of rhodamine (Rh123), an indicator of MDR activity, in MCF-7/DOX cells, and this suggests an accumulation of Rh123 in MCF-7/DOX cells, which mediated ABC transporters inhibition and drug efflux suppression through the blocking of P-glycoproteins (P-gp)-mediated MDR [217]. In cisplatin-resistant human ovarian cancer cells, β-elemene inhibited DNA repair by blocking cisplatin-mediated PI3K/JNN and PI3K/Akt activation [218]. Additionally, β-elemene increases the accumulation of doxorubicin and Rh123 in human gastric adenocarcinoma cells. This activity was suggested due to the inhibitory effect of β-elemene on P-gp, down-regulation of Akt and overexpression of E3 ubiquitin ligases [219].

The sensitization effect of auraptene was also assessed on growth and sphere formation (surrogate tumors) of colorectal adenocarcinoma wild type (HT-29 and HT-116 cells) and FOLFOX (chemotherapy combination of folic acid+ 5-fluorouracil, oxaliplatin)-resistant HT-29 and HT-116 cells. Auraptene significantly decreased the tumor markers of colon cancer (pEGFR, CCD44, CD166) only in HT-29 FOLFOX-resistant cells. The effect of auraptene on colon sphere formation size and tumor integrity was also assessed and showed a 40% reduction in the number of colon spheres in HT-29 FOLFOX-resistant cells [220]. As a future consideration, it is important to understand the link between the synergistic effect of auraptene, cell cycle control and apoptosis before introducing this bioactive compound into clinical studies.

Perillic acid was detected as a radiosensitizer in chemoradiation therapy of head and neck cancer cells. The molecular mechanism through which perillic acid exerted its radiosensitizer effect is not fully understood; however, the suggested mechanism of action was by triggering apoptosis [221].

The overactivation of NF-_k_B, a transcriptional factor, is associated with cisplatin-resistant development in several cancer cells [222]. Inhibition of the translocation of NF-_k_B by thymoquinone in a synergistic study with cisplatin resolved cisplatin resistance in non-small-cell lung cancers (NCI-H460) [223]. On the other hand, cisplatin-induced nephrotoxicity is one of the limitations associated with the clinical application of this drug. To resolve this limitation, thymoquinone was tested in an in vivo study, as a result, it exerted its protective effect via playing different roles, including antioxidant, calcium antagonist and vasodilatory [224]. Moreover, thymoquinone was reported to inhibit the nephrotoxicity effect of ifosfamide, a chemotherapeutic drug, and enhance its anticancer activity [225].

In pancreatic cancer cells, thymoquinone chemosensitizer cells to gemcitabine and oxaliplatin. The chemosensitization molecular mechanism of this compound was via triggering down-regulation of the transcription factor, NF-_k_B, Bcl-2 genes and NF-_k_B-dependent anti-apoptotic genes [85]. Thymoquinone in resistant MCF-7/DOX cell line enhanced cell death via up-regulating PTEN protein expression and the suppression of Akt phosphorylation as a cell survival regulator. Furthermore, this has triggered overexpression of p53 and p21 proteins accompanied by G_2_/M phase arrest [226]. A couple of studies revealed that nanoparticle encapsulation of thymoquinone improved the chemosensitizing activities of thymoquinone [227,228].

In another study, artesunate sensitized castrate-resistant prostate cancer cells (PC3, 22RV1, LNCaP) to antigen receptor antagonists and abrogated oncogenesis by suppressing NF-_k_B signaling. Artesunate enhanced cell death in cancer cells as a result of increased ROS level and oxidative stress. A possible sensitization effect of artesunate could be due to the presence of a high concentration of intracellular iron in the form of ferritin in cancer cells. Iron binds to transferrin (iron transporter protein) and forms holo-transferrin, which binds to the transferrin receptor and is internalized into cancer cells through receptor-mediated endocytosis. Prostate cancer cells showed an enhanced level of transferrin receptor. Hence, this was suggested as a plausible sensitization effect of artesunate in prostate cancer cells. The findings of this study suggested the necessity for clinical trials to test artesunate in combination with androgen receptor antagonists in castrate-resistant prostate cancer patients [229].

In another study, artesunate induced intrinsic apoptosis in doxorubicin-resistant leukemia cells (J16) through the mitochondrial pathway, which involved the release of cytochrome *C* from mitochondria and the activation of caspase 9. This activity was attributed to the generation of ROS induced by artesunate. Therefore, it was suggested that artesunate has the potential to sensitize cells to doxorubicin to trigger apoptotic cell death. This is because artesunate and doxorubicin use various killing mechanisms, where artesunate kills cancer cells via ROS generation, while doxorubicin kills cells via the inhibition of DNA polymerases, DNA topoisomerase II and DNA methyltransferase. The different cell inhibitory mechanism of artesunate highlights its application in combination with other established anticancer agents that induce cell death via a pathway different than artesunate [230].

**Figure 15:**
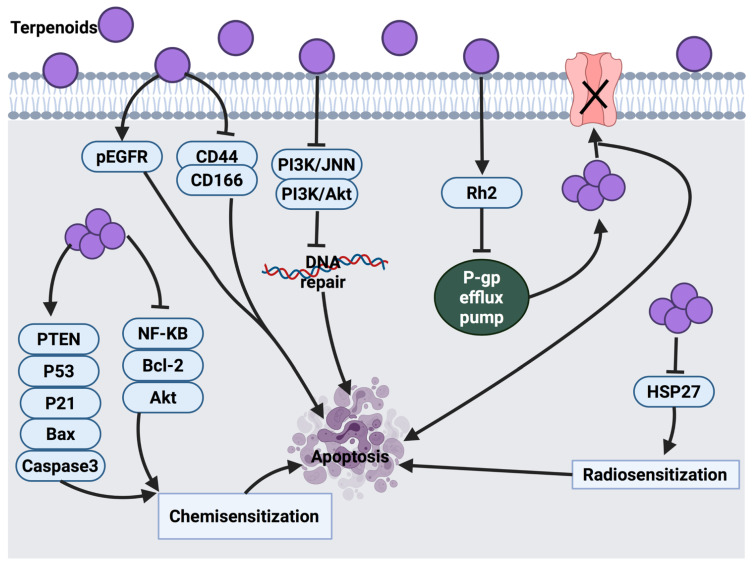
Mechanisms involved in the sensitization of cancer cells to chemotherapy by certain terpenoids (β-elemene, auraptene, perillic acid, thymoquinone, artesunate and triptolide). PTEN: phosphatase and tensin homolog, p53: tumor protein, pP21: cyclin-dependent kinase inhibitor 1, Bax: Bcl-2 associated X protein, NF-_K_B: nuclear factor light chain enhancer of activated B cells, Bcl-2: B-cell lymphoma 2, pEGFR: phosphorylated epidermal growth factor receptor, CD44: homing cell adhesion molecule, CD166: cluster of differentiation 166, PI3K/Akt: phosphatidylinositol 3-kinase/protein kinase B, Rh2: rhodopsin 2, P-gp: P-glycoprotein 1, HSP27: heat shock protein 27.

The sensitization effect of triptolide was investigated in cancer cells with a poorer prognosis, such as androgen-sensitive prostate cancer cells. The mechanism of action of this compound involved the reduction of androgen receptors and inhibition of the downstream targets, such as prostate-specific antigen (PSA) and NKX3.1 prostatic tumor suppressor gene. Further investigations showed that triptolide reduced the transcription activity of androgen receptors by inhibiting Sp1 (transcription factor) and HSP70 expression [231]. In addition, the sensitization effect of triptolide on pancreatic cancer cells toward gemcitabine was suggested through mitochondrial-induced apoptosis and inhibition of HSP27 [186]. Liu et al. [187] reported that lupeol sensitized human gastric carcinoma cells (SGC7901 and BGC823) to 5-FU. The suggested mechanism of action was through induction of apoptosis by up-regulation of Bax and p53. Figure 15 summarizes the molecular mechanisms involved in the sensitization effect of some terpenoids.

## 5. Clinical Trials and Patents of Certain Terpenoids as an Anticancer Agent

Few clinical trials were performed to investigate the anticancer therapeutic effect of some terpenoids. A phase I clinical trial on the combination of D-limonene and 5-FU revealed a positive response in a patient with breast cancer and over six months steady disease condition in three colorectal cancer patients [232]. An open-label pilot clinical study showed that 2 g of D-limonene induced changes in systemic and tissue biomarkers of breast cancer risk or carcinogenesis in 43 recruited women with newly diagnosed operable breast cancer. The researchers reported that D-limonene reduced the expression of cyclin D1, which may lead to cell-cycle arrest and reduced breast cancer cell proliferation [232]. A recent clinical trial study investigated the anticancer effect of a few terpenoids, with β-elemene being the most promising terpenoid. In this study, β-elemene was tested in patients with advanced non-small-cell lung cancer (NSCLC), advanced lung cancer, lung cancer with brain metastases, lung cancer in the older patient with EGFR mutant, advanced gastric cancer, esophageal cancer, liver cancer, colon cancer, breast cancer, cervical cancer, nasopharyngeal cancer, leukemia, lymphoma, brain tumor, ovarian cancer and endometrial cancer. The outcomes of this study showed a high median survival rate and tumor control rate with significantly fewer side effects [233]. A single-center randomized, double-blind placebo-controlled trial study showed that 14 daily doses of oral artesunate (200 mg) exerts antiproliferative properties in colorectal cancer via reducing Ki67, a protein that the up-regulation of which is associated with a poorer prognosis in colorectal cancer patients and increasing CD31 expression [234]. Moreover, another study showed that intravenous artesunate (18 mg/kg) is well tolerated and exerts modest clinical activity in patients with advanced solid tumor malignancies [235]. It is worthy of mention that structural modification of terpenoids is likely to reveal a new strategy in developing analogs that might enhance the bioavailability and efficacy, and therefore, could be considered in human clinical trials [235]. There are a few more terpenoids currently under different phases of clinical trials, and the details of these RCTs are summarized in Table 3.

We used a specialized patent database WIPO (World intellectual property organization) to search for the patented terpenoids as anticancer agents. A total of 23 patents were identified during our search, 13 of them were patented in China, while the rest were registered in different countries, including the USA, India, Japan, Singapore, Russia and European countries (Table 4). All these patents emphasize the potential role of terpenoids as anticancer agents for different types of cancer.

## 6. Current Challenges and Future Perspective for Using Terpenoids as Anticancer Agents

Terpenoids, a family of secondary metabolites with a wide range of chemical structures and biological activities, are a promising source for finding new anticancer agents Several investigations have found that this type of secondary metabolite has a cancer-fighting effect. Some of these molecules, such as D-limonene, are well tolerated by patients and have low toxicity, making them suitable for clinical use in the near future [246]. On the other hand, some sesquiterpene lactones, such as parthenolide, are unsuitable for clinical use due to their off-target effects and limited bioavailability [247]. However, parthenolide may serve as a lead compound for the development of synthetic compounds with better specificity and favorable pharmacokinetic properties. Some of the semisynthetic diterpenoid taxanes are already approved for cancer management and are currently widely used as chemotherapeutic agents, such as paclitaxel and docetaxel. Other novel taxanes are being developed to lower their toxicity and boost efficacy, which acts by inhibiting microtubules, inducing mitotic arrest and overcoming cell resistance [248]. The pharmacological activity of these compounds is intriguing; for example, some diterpenoids present a lead structure for developing a dual modulator of androgen and estrogen receptors for use in hormone-responsive cancer chemoprevention and/or chemotherapy [247].

The hydrophobic property of terpenoids has a significant effect on the drug’s bioavailability and limits its clinical use [249]. Therefore, producing more hydrophilic derivatives of terpenoids may boost its pharmacokinetic features, such as oral bioavailability and plasma concentration. Furthermore, a terpenoid’s toxicity can be reduced by structural alterations, such as reduced epoxidation or oxidation of the allylic methyl groups [250,251,252,253]. Studies have shown that terpenoids that have at least one N-methyl group exert higher anti-leukemic action than those without. Fluorinated terpenoid derivatives showed increased cytotoxicity against multiple myeloma. In addition, studies have shown that dimethylamino parthenolide is more water-soluble and orally bioavailable compared to parthenolide, which has a 70% oral bioavailability in rats [254].

Terpenoids extracted from *Curcumae rhizoma* essential oil are promising agents for cancer therapy and are well-known as possible anticancer agents. For instance, β-elemene has been developed into a novel drug for treating solid tumors in China, and it is presently being tested in clinical trials in the United States. There is no doubt that β-elemene, as the bioactive non-curcuminoids compound in *Curcumae rhizoma*, is a promising anticancer agent with a broad-spectrum anti-tumor action, minimal constitutional toxicity and ease of administration, as revealed by numerous in vitro and in vivo investigations [233]. However, the therapeutic application of β-elemene was further hampered by its poor solubility, low absorption and various adverse responses (e.g., phlebitis, fever, discomfort, drug extravasation resulting in necrosis and local inflammation). As a result, studying the novel characterization system of β-elemene is extremely important from a scientific standpoint. At the moment, drug-loaded β-elemene characterization systems are loosely split into four categories: liposomes, solid lipid nanoparticles, microemulsions and microcapsules, all of which are still in the development stage. Thymoquinone encapsulation in a liposome is an example of the successful application of this strategy, which resulted in increased stability and bioavailability of this terpenoid while maintaining its anticancer action [255]. Furthermore, new technologies, such as biochip, microarray and proteome, are powerful tools to search for specific and selective targets for β-elemene and its derivatives in the future [233].

Changes in gene regulation and expression are influenced by epigenetic modifications ranging from DNA methylation to histone modification and microRNA-mediated modification. A new cancer prevention technique involves dietary factors impacting epigenetic alteration to turn on/off gene expression and signaling pathways. Many bioactive dietary components, such as triterpenoids, have recently received a lot of attention because of their effects on epigenetic alterations and epigenome profiles, which could help prevent cancer. However, research into the interplay between phytochemicals and epigenetic/epigenomic machinery in cancer prevention and health is still in its infancy [256]. Future research could concentrate on understanding the in vivo mechanisms, identifying epigenetic regulatory switches, developing new analogs and enhancing the bioavailability of triterpenoids to aid in the development of more effective agents for cancer management.

Before introducing a novel anticancer drug to clinical trials, preclinical studies are necessary. However, there is a gap in the link between preclinical data and clinical outcomes of terpenoids. Despite large sums of money spent on preclinical settings for target selection, validation and lead optimization, the majority of terpenoids fail in clinical trials. This could be because the models utilized in preclinical settings are not good enough to accurately mimic human responses. Cell lines, to a certain extent, mimic oncogenic changes found in tumors, such as somatic mutations, DNA methylation and gene expression. Cancer cell lines are extensively used for testing new cancer treatments, including terpenoids [257]. However, there are some drawbacks and challenges to this paradigm. For example, genomic instability can cause differences between the original tumor and the cell line, culture conditions may change the morphology, gene expression pattern, genomic profile, cellular pathways and culture environment from the original tumor, and natural tumor heterogeneity can be lost [258,259]. Furthermore, cancer cells lack a complex extracellular matrix and proliferate in the absence of stroma, which includes lymphatic arteries and blood, as well as related fibroblasts and immune cells. As a result, there is frequently a fundamental mismatch between in vitro data and clinical findings, which can be viewed as one of the primary reasons for innovative drug development failure [260]. Therefore, using only cells from a single origin to evaluate the efficacy of novel terpenoids is insufficient; instead, a diversity of cancer cells from various origins, including those of human origin, should be used. Various animal models, such as athymic nude mice, xenograft, orthotopic and zebrafish, are widely used in cancer research [261]. However, these animal models cannot accurately reflect all aspects of human cancer [262]. Recent improvements in in vitro 3D culture techniques, such as organoids, have opened up new possibilities for developing more physiological and unique human cancer models for translating fundamental cancer research into novel therapy options for cancer patients. Future studies on the anticancer effect of terpenoids can apply 3D culture techniques to overcome the limitations of other models.

The lack of robustness during the preclinical stage of terpenoids screening and investigating the underlying anticancer mechanism of action is mainly the reason for the failure of most terpenoids in clinical trials. In preclinical models, terpenoids were studied in a fairly limited set of experimental conditions. As a result, when terpenoids are finally tested in cancer patients, the challenges are posed by the complex genetic makeup of the human population and the complexities and heterogeneity of cancer. The utilization of the most recent breakthroughs in molecular, cellular and chemical structural modification techniques, as well as high-throughput screening of terpenoids is expected to lead to more robust preclinical testing and, as a result, more promising outcomes during clinical trials.

## 7. Conclusions

Terpenoids as bioactive compounds derived from natural sources are potential agents for cancer prevention. Certain terpenoids showed synergistic effects in combination with doxorubicin, cisplatin, paclitaxel, sorafenib and 5-fluorouracil, especially against multidrug resistant cancer cells; therefore, some terpenoids are considered as effective chemosensitizers that may enhance the response to conventional chemotherapy. Terpenoids mainly exert their anticancer effects by targeting various pathways, including mitochondrial death pathway, PI3K/Akt and NF-_K_B pathways. Several experimental pieces of evidence encourage the development of terpenoids as drugs for the treatment and prevention of cancer and other inflammatory diseases. However, one major limitation on gaining notable efficacy of terpenoids is due to their poor absorption and low bioavailability. Hence, structural modification of terpenoids is necessary to reveal new strategies for developing analogs that might enhance the bioavailability and efficacy. Considering the effectiveness of terpenoids, future studies should be centered on comprehensive preclinical toxicity, bioavailability, pharmacodynamics, biomarkers and wide investigations of tumor suppression using appropriate animal models before going into extensive clinical studies.

## Figures and Tables

**Table 1:** List of the terpenoids reported in this review that exhibit sensitizing effects and therapeutic potential for cancer treatment.

Classification	Terpenoids
Monoterpenoids	Thymol [10,11,12,13,14,15,16,17,18,19], Menthol [20,21,22,23,24,25], Auraptene [26,27,28,29,30,31,32,33,34], D-limonene [35,36,37,38,39,40], Perillic acid [41,42], Ascaridole [43,44], Carvacrol [45,46,47,48,49,50,51,52,53,54,55,56,57,58,59,60,61,62], Thymoquinone [54,63,64,65,66,67,68,69,70,71,72,73,74,75,76,77,78,79,80,81,82,83,84,85,86,87,88,89,90,91,92,93,94,95,96,97,98]
Sesquiterpenoids and Sesquiterpene lactones	Ambrosin, coronopilin, and dindol-01 [99], Parthenolide [100,101,102,103,104], Costunolide [105], Dehydrocostuslactone [106], Helenalin [107,108,109,110], EM23 [111], Artesunate & Artemisinin [112,113,114,115,116,117,118,119,120,121,122,123,124,125,126,127,128,129,130,131,132,133,134,135,136,137], β-Elemene [138,139]
Diterpenoids	Triptolide [140,141,142,143,144,145,146,147,148,149,150,151,152,153,154], Crocetin [155,156,157,158,159,160,161,162,163,164,165], Phytol [166,167,168]
Triterpenoids	Ursolic acid [169,170,171,172,173,174,175,176,177,178,179,180], Betulinic acid [181,182,183,184,185,186,187,188,189,190,191,192,193,194,195,196], Lupeol [60,61,197,198,199,200,201,202,203,204,205]

**Table 2:** Classification of terpenoids.

Class	Number of Carbon Atoms
Monoterpenoids	C_10_
Sesquiterpenoids	C_15_
Diterpenoids	C_20_
Triterpenoids	C_30_

**Table 3:** List of some terpenoids as anticancer agents under various phases of clinical trials.

Terpenoids	Condition	Sample Size	Status	Phase	NCT Number	Reference
Menthol	Colon cancer	60	Unknown	2	NCT01855607	[236]
	Esophageal cancer	648	Unknown	3	NCT02355249	[237]
	Gastric cancer	85	Completed	3	NCT01411176	NA
	Gastric cancer	33	Completed	3	NCT01411189	NA
	Cervical cancer	3612	Completed	NA	NCT02255084	[238]
	Blood and breast cancer	585	Completed	2	NCT00962494	NA
D-limonene	Breast cancer	59	Completed	1	NCT01046929	NA
	Breast cancer	103	Completed	1	NCT01459172	[239]
	Submandibular gland tumor and parotid gland tumor	10	Completed	1	NCT04296266	NA
	Pancreatic cancer	21	Completed	1	NCT02336087	NA
Thymoquinone	Oral potentially malignant lesion	48	Completed	2	NCT03208790	[240]
Artesunate	Colorectal and bowel cancer	200	Active, not recruiting	2	NCT02633098	NA
	Colorectal cancer	200	Recruiting	2	NCT03093129	[241]
	Metastatic and locally advanced breast cancer	23	Completed	1	NCT00764036	[242]
	Solid tumor	19	Completed	1	NCT02353026	[243]
	Lung cancer	30	Unknown	1/2	NCT02786589	[244]
	Hepatocellular carcinoma	2	Completed	NA	NCT02304289	NA
β-elemene	Non-small-cell lung cancer	80	Unknown	2	NCT03123484	[245]
Triptolide	Pancreatic cancer	19	Completed	2	NCT03117920	NA
	Advanced, gastric, breast, pancreatic, prostate, colorectal cancers, solid tumor, solid carcinoma, solid carcinoma of stomach and cancer of stomach	66	Recruiting	1	NCT03129139	NA
	Adenosquamous carcinoma of pancreas	55	Recruiting	2	NCT04896073	NA

Source: https://clinicaltrials.gov/; accessed on 22 October 2021.

**Table 4:** List of patented terpenoids as anticancer agents.

Terpenoids	Condition	Patent No.	Country
Thymol	Cancer	1020200023123	China
	Oral cancer	106691931	China
Menthol	Cervical cancer	108652091	China
	Cancer	1082337	China
D-limonene	Cancer	201941035781	India
	Cancer	106890140	China
Sesquiterpene lactone	Liver cancer	110585194	China
	Cancer	02233661	Russian Federation
β-elemene	Cancer	101165037	Chin
	Cancer	101200448	China
Triptolide	Liver and breast cancer	104327152	China
	SCLC	103393598	China
	Breast and triple negative breast cancer	103405443	China
	Mesothelioma	3348276	European patent office
	Mesothelioma	WO/2017/043613	Japan
	Mesothelioma	11201802915W	Singapore
	Mesothelioma	108025070	China
	Mesothelioma	201827013711	India
	Mesothelioma	20180326052	United States of America
Phytol	Cancer	WO/2017/131175	Japan
	Liver cancer	201941045086	India
	Cancer	20190343909	United States of America
	Cancer	108601756	China

Source: https://patentscope.wipo.int/search/en/search.jsf; accessed on 28 October 2021.

## Data Availability

Not applicable.

## Supplementary Materials

The following supporting information can be downloaded at: https://www.mdpi.com/article/10.3390/cancers14051100/s1, Table S1. Effective concentrations of certain terpenoids and their targets based on IC_50_ values or tested range of concentrations against different cancer cell lines.
