# Supplementary material for: Therapeutic Potential of Certain Terpenoids as Anticancer Agents: A Scoping Review
Source: Cancers (Basel). 2022 Feb 22;14(5):1100. doi: 10.3390/cancers14051100 (PMC8909202; doi:10.3390/cancers14051100)
Supplement: Supplementary file 1 [file cancers-14-01100-s001.zip › cancers-1563180-supplementary.pdf]

cancers MDPI

Supplementary Materials: Therapeutic Potential of Certain Terpenoids as Anticancer Agents: A Scoping Review

Sareh Kamran, Ajantha Sinniah, Mahfoudh A. M. Abdulghani and Mohammed Abdullah Alshawsh *

Table S1. Effective concentrations of certain terpenoids and their targets based on IC_{50} values or tested range of concentrations against different cancer cell lines.

Terpenoid	Cell Line	IC_{50} or Tested Range of Concentration	Target
Thymol	HL-60	25 and >50 µM	AP1, NFAT-2, IL-2, IFN-γ, ROS, caspase 9 & 3, Ca^{2+}
	AGS	100-400 µM	
	T24, SW780, J82	100 µM	
	OSCC	>4.3 mM	
	JM	50 µg/mL	
	MG63	600 µM/L	
	PC3	900 µM	
	DBTRG-05MG	200-600 µM	
Menthol	SNU-5	1.62 mg/L	Topoisomerase, p21, Bcl-2, caspase 9 &3, TRPM8, Ca^{2+}
	LNCap	3.2 mM/L	
	T24	1000-2000 µM/L	
	DU145	75-100 µM/L	
	PC3	2 µM/L	
Auraptene	SNU-1	25-200 µg/ml (plant extract with auraptene as a major compound)	MMP-2, MMP-9, TNF receptor, P-gp, Bcl-2, Mcl-1, caspase 9 & 3, ERK1/2, proinflammatpry cytokins, PCNA, CSC markers, p53, p21, mTOR, 4E-BP1, S6
	KYSE30	20 µg/Ml	
	HeLa cells	25-100 µM/L	
	A2780	6.25-100 µM/L	
	Caco-2 & DLD-1	75 µM/L	
	MCF-7	10 µM/L	
	PC3 & DU145	15-90 µM/L	
D-limonene	HL-60	0.75 mM/L	NO , Bcl-2, p53, Bcl-2, ROS, caspase 9 & 3, PI3K, PDK1, Akt, Ras, Raf, MEK, ERK
	A549	0.5 mM/L	
	H1299	0.75 mM/L	
	MGC803	182 µM/L	
	LS174T	3.2 mM/L	
Perillic acid	A549	3.6 mM/L	Apiptosis, cell cycle arrest
	HCT116	0.75 mM/l	
Ascaridole	XP3BE	0.18 µM/L	Cell cycle arrest, oxidative DNA damage, apoptosis
	GM10902	0.15 µM/L	
	HL-60	6.3 µg/Ml	
	HCT-8	18.4 µg/mL	
	SF-295	8.4 µg/mL	
	MDA-MB-435	10.5 µg/mL	
Carvacrol	HCT116	544.4 µM/L	Ras, Natch-1, Jagged-1, IL-6, pSTAT3, pERK1/2, pAkt, ROS, MAPK, PI3K/Akt,
	LoVo	530.2 µM/L	

	AGS	82.57 µM/L	Bax, p53, pJNK, caspase 9 & 3, Bcl-2, pAkt, Cyclin B, CDK-1, CDK-4
	MCF-7	244.7 µM/L	
	HepG-2	0.4 µM/L	
	A549	250-1000 µM/L	
	LNCaP	135 µM/L	
	MDA-MB-231	100 µM/L & 199 µM/L	
	U87	322 µM/L	
	PC3	360 µM/L	
	Hep-2	0.2 mM	
	DU145	500 µM/L	
	CO25	60 µg/mL	
	P815	0.004% v/v & 1.2% v/v	
	CEM	1.2% v/v	
	K-562	1.2% v/v	
	MCF-7	2.5% v/v	
	MCF-7 gem	0.85% v/v	
Thymoquinone	Hep-2	22.2 µM/L	$CD8^+T$, cd62L, QR, GSH, p16, CDK-1, p53, CHEK-1, GSK-3, p73, NF-κB, XIAP, Bax, caspase 8, 9 & 3, IL-6, STAT3, JAK, SRC, MAPK, PI3K/Akt, ERK, JNK, Wnt, ALP, AIF, Gadd45α, Bcl-2, MUC4, p38
	A549	2.1 µg/mL	
	DLD-1	1.0 µg/Ml	
	U87MG	38.82 µM/L	
	T98G	62.48 µM/L	
	NCI-H460	80 µM & 100 µM	
	KBM-5	25 µM/L	
	HCT-116	60 µM/L	
	U266 and RPMI8226	20 µM/L	
	MG63	17 µM/L	
	SaOS-2	20-80 µM/L	
	HeLa	5.37 µg/mL	
	HT-29	110 µM/L	
	HCT-116	14 µM/L	
	DLD-1	23 µM/L	
	LoVo	28 µM/L	
	Caco-2	12.5 µM/L	
	C4-2B	50 µM/L	
	PC-3	80 µM/L	
	HL-60	23 µM/L	
	HepG2	350 µM/L	
	SP-1	30 µM/L	
	17 cells	60 µM/L	
	T-47D	18.06 µM/L	
	MDA-MB-468	12.30 µM/L	
	M059K and M059J	50 µM/L	
	Neuro-2a	36 µM/L	
	FG/COLO357	73 µM/L	
	LNM35	50-78 µM/L	
	HepG2	34 µM/L	
	HT29	50-78 µM/L	
	MDA-MB-435	50-78 µM/L	

	MDA-MB-231	50-78 µM/L	
	MCF-7	50-78 µM/L	
	T28	30 µM/L	
	A431	10 µM/L	
	Hep-2	40 µM/L	
	RPMI 2650	2650 µM/L	
	U87	100 µM/L	
	Jurkat cell	50 µM/L	
	T98, LNCaP, and 3T3	10-50 µM/L	
	BFTC909, 786-O	60-80 mM/L	
	786-O-SI3	40-80 mM/L	
	ACHN	72 µM/L	
	786-O	55 µM/L	
Ambrosin	MCF-10A<br>MCF-7<br>JIMT-1<br>HCC1937	2.1 µM/L<br>1.7 µM/L<br>1.4 µM/L<br>4.1 µM/L	p53, NF-κB
Coronopilin	MCF-10A<br>MCF-7<br>JIMT-1<br>HCC1937	15 µM/L<br>16 µM/L<br>5.5 µM/L<br>16 µM/L	p53, NF-κB
Dindol-01	MCF-10A<br>MCF-7<br>JIMT-1<br>HCC1937	37 µM/L<br>16 µM/L<br>16 µM/L<br>15 µM/L	p53, NF-κB
Sesquiterpene lactone extracted from *Artemisia macrocephala*	3T3, HeLa, MCF-7	500 & 1000 µg/mL	NF-κB
Parthenolide	HepG2, Hep3B, SK-Hep1; HepG2/STAT3, MEF, HEK293, Hela, Hs578t, HBE, H4, MDA-MB-453, MDA-MB-231, MDA-MB-468, HCT116, NCI-H1975, Du145	15 µM/l; 10 mM/L	ER, Ca2+, TAC, P53, IKba, NF-KB, JAK2, IL-6, STAT, ROS generation
Costunolide	SW-872, SW-982, TE-671	10 µg/mL	cyclin D1, cyclin D3, CDK4, CDK6, p18 INK4c, P21 CIP/Waf-1, p27 KIP1, FasL, TNF-α
dehydrocostus lactone	HepG2, PLC/PRF/5	30 µM/L	Bax, Bak, Bcl-2, Bcl-XL, AIF, Endo G
Helenalin	A2780	2 µM/L	hTERT, mitochondrial apoptosis, NF-κB, P65
EM23	K562; HL-60	10.8 µM/L; 1.9 mM/L	Trx, ASK1, p38, JNK, ERK MAPKs
Artesunate & artemisinin	MG-63 and U20S<br>HNC<br>HCT-116<br>CLY	75 µM/L<br>100 µM/L<br>40 & 80 µg/mL<br>20 µM/L	PCNA, Bcl-2, Bcl-xL, c-Fos, KLF6, Bax, P21, P53, fas, CDC25A, Caspase3 and 9, GSH, ROS, Keap1, P62, PAX7, β-catenin,

	SK-BR-3, MDA-MB-468, MCF-7 and MDA-MB-231	25-100 μM/L	TCF/LEF, Nrf2-ARE, PTEN, miR-200c, Hotair, Cox2, EGFR, Akt, ABCG2, HSP20, HSP27, ERK, STAT3, STAT5, P38, CREB, VEGF, JAK-STAT, Cytc, Caspase3
	GH3	9.53 μM/L	
	MMQ	18.37 μM/L	
	A549	25-100 μM/L	
	TE671	50 μM/L	
	WERI-Rb1	10-40 μg/mL	
	PANC-1 & CFPAC-1	100-200 μM/L	
	PLC/PRF/5	121.2 μM/L	
	HuH7	46.4 μM/L	
	HepG2	37.4 μM/L	
	Hep3B	41.6 μM/L	
	HCCLM3	44.2 μM/L	
	HepG2	20 μM/L	
	Eca109	30-120 μM/L	
	SGC-7901	78.2 μM/L	
	BGC-823	72.3 μM/L	
	AGS	102.3 μM/L	
	A431	60 μM/L	
	HepG2	50 μM/L	
	HaCaT	120 μM/L	
	PC3 & 22RV1	>70 μM/L	
	LNCaP	4.42 μM/L	
	K562	12.5-100 μg/mL	
	U937	3.125-100 μg/mL	
	J16	2 μg/mL	
β-elemene	H460; A549	42 μg/mL; 48 mg/mL	P27, CDK1, cyclin B1, Chk2, CDC25C, CDK1, Cyclin A-CDK2 complex, p27, cyclin-A, cyclin B1, Bcl-2
Triptolide	KB	4.41 nM/mL	HMGB1, PI3K, Akt, mTOR, LC3B-II, p62, CD31, CD105, IL-2, TNF-α, CD16, CD56, caspase 9 & 3, glucose, HKII, GSH, Nrf2, FAK, ERK1/2, MDM2, P70S6K, XBP1, Rpb1, Cdk7, p44, RNA polymerase II, itgb1, mmp-2, mmp-9
	KB-7D	8.1 nM/mL	
	KB-tax	4.52 nM/mL	
	H460, A549, H358	10 nM/L	
	H1299 & NCI-H460	100-150 nM/L	
	MCF-7 & MDA-MB-231	5-160 nM/L	
	SiHa	25-100 nM/L	
	MDA-MB-231	Under normal serum condition: 0.3 nM/L and in serum free condition: 12.7 nM/L	
	SKOV/DDP	8 ng/mL	
Crocetin	KYSE-150	125.812 μM/L	ABCB1, ABCC1, DNA & RNA polymarease II, EFGR, pCdc25c, p-Cdc-2, cyclin B1, TDP1, caspase 9 & 3, p53, p21, Bax,
	APL, HL60 & NB4	50-100 μM/L	
	AGS	50 μM/L	
	SKOV3	119.76 μM/L	
	HeLa	119.32 μM/L	
	A549	101.34 μM/L	
Phytol	A549	60.7 μM/L	

	AGC	147.67 µM/L	Bcl-2, Bax, caspase 9 and 3, PARP, LC3-I, LC3-II, Akt, mTOR, p70S6K, N-acetyl-L-cystein
	MCF-7	8.79 µM/L	
	MDA-MB-231	69.67 µM/L	
	HeLa	15.51 µM/L	
	PC-3	77.85 µM/L	
	HT-29	34.82 µM/L	
	A-549	56.98 µM/L	
	Hs294T	65.15 µM/L	
	MRC-5	124.84 µM/L	
Ursolic acid	HaCaT & M4Beu	12.5-15 µM/L	Ca^{2+} , cytochrom *C,* Akt , HK2, pyruvate kinase M2, autophagy, Fas, caspase-8, PARP, Bax, Bcl-2, caspase-9 and 3, p53
	HuH7	75 µM/L	
	U-2OS	28.3 µM/L	
	MG-63	27.2 µM/L	
	MDA-MB-231	40 µM/L	
	SW480	8 µM/L	
	B16F-10	75-100 µM/L	
	SMMC-7721	40 µM/L	
Betulinic acid	UISO-Mel-1	8 µg/mL	Bcl-2, Bcl-xL, Bax, Bad, Apaf-1, caspase 9 & 3, ROS, MAPK, p38, p53, p21, IKKβ, IKKα, NF-κB, cyclin D1, cyclin B1, EGFR
	A2780	1.8 µg/mL	
	OVCAR-5	3.3 µg/mL	
	IGROV-1	4.5 µg/mL	
	H460	1.5 µg/mL	
	A431	1.8 µg/mL	
	Me665/2/21	1.5 µg/mL	
	Me665/2/60	1.6 µg/mL	
	POGB	4.2 µg/mL	
	POGB/DX	3.2 µg/mL	
	C18161	1.5 µg/mL	
	HeLa	10 µg/mL	
	HepG2	10.1 µg/mL	
	A549	14.8 µg/mL	
	MCF-7	13.6 µg/mL	
	NCl-H460	28.1 µg/mL	
	PC-3	36.7 µg/mL	
	SK-HEP-1	58.5 µg/mL	
	K562	>100 µg/mL	
	CL-1	23.50 µM/L	
	CLBL-1	18.2 µM/L	
	D-17	18.59 µM/L	
	HeLa	30.42 µM/L	
	H460	50 µM/L	
Lupeol	MCF-7	80 µM/L	PI3K, PDK1, Akt, p53, Bax, EGFR, IKβ, NF-κB, ROS, Apaf-1, caspase 9 & 3, Wnt, CLDN1, CCNA2, Ras, Raf, MEK, ERK, STAT3
	A549	62.53 µg/mL	
	PC3	500 µM/L	
	GBC-SD	46 µM/L	
	Mel-928	75 µM/L	
	Mel-1241	72 µM/L	
	Mel-1011	135 µM/L	

SW480	90.2 µM/L
HCT-116	53.3 µM/L
AsPC-1	35 µM/L
UPCI:SCC131	26.1 µM/L
UPCI:SCC084	21.42 µM/L
H1299 & A549& H460	100 µg/Ml
HeLa	50-100 µM/L
SiHa	25-100 µM/L
